# Bicyclol attenuates pulmonary fibrosis with silicosis via both canonical and non-canonical TGF-β1 signaling pathways

**DOI:** 10.1186/s12967-024-05399-x

**Published:** 2024-07-26

**Authors:** Tong-Tong Liu, Hai-Fei Sun, Ming-Ze Tang, Hao-Ran Shen, Zhen Shen, Yan-Xing Han, Yun Zhan, Jian-Dong Jiang

**Affiliations:** State Key Laboratory of Bioactive Substance and Function of Natural Medicines, Institute of Materia Medica, Chinese Academy of Medical Sciences and Peking Union Medical College, Beijing, 100050 People’s Republic of China

**Keywords:** Bicyclol, Silicosis, Pulmonary fibrosis, TGF-β1, JAK2/STAT3, SMAD2/3, EMT, FMT

## Abstract

**Background:**

Silicosis is an irreversible fibrotic disease of the lung caused by chronic exposure to silica dust, which manifests as infiltration of inflammatory cells, excessive secretion of pro-inflammatory cytokines, and pulmonary diffuse fibrosis. As the disease progresses, lung function further deteriorates, leading to poorer quality of life of patients. Currently, few effective drugs are available for the treatment of silicosis. Bicyclol (BIC) is a compound widely employed to treat chronic viral hepatitis and drug-induced liver injury. While recent studies have demonstrated anti-fibrosis effects of BIC on multiple organs, including liver, lung, and kidney, its therapeutic benefit against silicosis remains unclear. In this study, we established a rat model of silicosis, with the aim of evaluating the potential therapeutic effects of BIC.

**Methods:**

We constructed a silicotic rat model and administered BIC after injury. The FlexiVent instrument with a forced oscillation system was used to detect the pulmonary function of rats. HE and Masson staining were used to assess the effect of BIC on silica-induced rats. Macrophages-inflammatory model of RAW264.7 cells, fibroblast-myofibroblast transition (FMT) model of NIH-3T3 cells, and epithelial-mesenchymal transition (EMT) model of TC-1 cells were established in vitro. And the levels of inflammatory mediators and fibrosis-related proteins were evaluated in vivo and in vitro after BIC treatment by Western Blot analysis, RT-PCR, ELISA, and flow cytometry experiments.

**Results:**

BIC significantly improved static compliance of lung and expiratory and inspiratory capacity of silica-induced rats. Moreover, BIC reduced number of inflammatory cells and cytokines as well as collagen deposition in lungs, leading to delayed fibrosis progression in the silicosis rat model. Further exploration of the underlying molecular mechanisms revealed that BIC suppressed the activation, polarization, and apoptosis of RAW264.7 macrophages induced by SiO_2_. Additionally, BIC inhibited SiO_2_-mediated secretion of the inflammatory cytokines IL-1β, IL-6, TNF-α, and TGF-β1 in macrophages. BIC inhibited FMT of NIH-3T3 as well as EMT of TC-1 in the in vitro silicosis model, resulting in reduced proliferation and migration capability of NIH-3T3 cells. Further investigation of the cytokines secreted by macrophages revealed suppression of both FMT and EMT by BIC through targeting of TGF-β1. Notably, BIC blocked the activation of JAK2/STAT3 in NIH-3T3 cells required for FMT while preventing both phosphorylation and nuclear translocation of SMAD2/3 in TC-1 cells necessary for the EMT process.

**Conclusion:**

The collective data suggest that BIC prevents both FMT and EMT processes, in turn, reducing aberrant collagen deposition. Our findings demonstrate for the first time that BIC ameliorates inflammatory cytokine secretion, in particular, TGF-β1, and consequently inhibits FMT and EMT via TGF-β1 canonical and non-canonical pathways, ultimately resulting in reduction of aberrant collagen deposition and slower progression of silicosis, supporting its potential as a novel therapeutic agent.

**Supplementary Information:**

The online version contains supplementary material available at 10.1186/s12967-024-05399-x.

## Introduction

Silicosis, a form of pneumoconiosis, is a serious occupational disease worldwide caused by long-time exposure to crystalline silica (CS) in workplaces. The disorder is characterized by silicon nodules and diffuse pulmonary fibrosis [1]. Due to its occult onset and continuous impairment of particle clearance, diagnosis of silicosis is difficult in the early stages until irreversible development into pulmonary fibrosis [2], which is accompanied by a sharp decline in pulmonary function that can lead to poor quality of life. Appropriate protection at work can help reduce the risk of silicosis. However, few effective therapeutic interventions other than lung transplantation are currently available that can delay or reverse fibrosis progression in patients with silicosis [3].

The formation and development of pulmonary fibrosis is a profoundly complex biological process. Inflammation is one of the most common causes of silicosis in which macrophages play an important role [4]. Alveolar macrophages (AMs) that recognize and engulf silicon dust are transported to the lung interstitium and differentiate into activated interstitial macrophages [5]. Subsequently, these macrophages undergo dysregulated polarization, with secretion of abundant cytokines such as IL-1β, IL-6, TNF-α, and TGF-β1, further promoting inflammation and progression of silicosis [6]. Among the inflammatory cytokines secreted, TGF-β1 is considered a central mediator of liver, kidney, and lung fibrosis [7]. TGF-β1 acts through a well-characterized canonical signaling pathway involving phosphorylation and activation of SMAD2 and SMAD3. Activated SMAD2/3, together with SMAD4, forms a complex that translocates to the nucleus and promotes transcription of specific genes [8]. In addition, TGF-β1 activates a variety of SMAD-independent pathways (designated ‘non-canonical signaling’) that function in modification of cell functions, including PI3K/AKT, Rho-like GTPase, and MAPK [9]. TGF-β1 stimulates terminally differentiated alveolar epithelial cells (AECs) to undergo epithelial–mesenchymal transition (EMT) and induces fibroblast-myofibroblast transition (FMT) of fibroblasts via both canonical and non-canonical signaling, resulting in activation of myofibroblasts, excessive production of extracellular matrix (ECM) and inhibition of ECM degradation [8, 10], and ultimately, pulmonary fibrosis and lung damage. Inhibition of any of these steps may present an effective strategy for treatment of silicosis.

Bicyclol (BIC), a synthetic class I hepatoprotective drug with proprietary intellectual property rights in China, has remarkable anti-hepatitis and anti-inflammatory effects [11]. Earlier research has shown that BIC improves abnormal liver function and plays a protective role against liver disorders, including prevention of hepatitis virus replication, reduction of liver damage, and inhibition of liver fibrosis [12–14]. The anti-fibrosis capability of BIC is not confined to liver disease. BIC is additionally reported to attenuate experimental fibrosis of different organs through inhibiting cell apoptosis and mitigating the overexpression of inflammatory cytokines, such as bleomycin-induced idiopathic pulmonary fibrosis [15], high-fat diet-induced myocardial fibrosis, [16] and renal fibrosis caused by unilateral ureteral obstruction [17], indicating its potential as a therapeutic candidate for fibrotic diseases. Previously, our group showed that BIC could partially reduce pulmonary fibrosis induced by silica, although its therapeutic efficacy and underlying mechanisms remain to be validated [18].

Here, we established murine in vitro and rat in vivo silicosis models to explore the therapeutic activity and mechanisms of action of BIC in silica-induced fibrosis. Notably, BIC improved pulmonary function, alleviated inflammatory cell and cytokine secretion and fibrosis in the lung of the rat model. Moreover, BIC protected macrophages from apoptosis and polarization and suppressed the secretion of inflammatory cytokines, in particular, TGF-β1. BIC suppressed TGF-β1-induced EMT in epithelial cells as well as FMT in fibroblasts via both canonical and non-canonical signaling pathways, resulting in inhibition of fibroblast migration and diminished ECM production. Our collective findings support the utility of BIC in clinical treatment of silicosis.

## Methods

### Reagents

Bicyclol (98% purity) purchased from Tai’an Jiangzhou Biotechnology Co., Ltd (Tai’an, China) was dissolved in 0.5% carboxymethyl cellulose sodium (CMC-Na).

Silicon dioxide (SiO_2_) particles (99% purity, diameter 0.5–5 μm) were obtained from Sigma-Aldrich (St. Louis, MO). A 100 mg/mL silica suspension was prepared using normal saline (NS) and autoclaved before use.

### Animal models

All research involving animals complied with protocols approved by the Animal Care and Use Committee of Peking Union Medical College. Male Wistar rats (4–6 weeks old, 180–200 g) were purchased from SPF (Beijing) Biotechnology Co., Ltd. (Beijing, China). Rats were randomly divided into the following three groups (*n* = 9): control, SiO_2_, and BIC. After anesthesia, rats were intratracheally instilled with 1 mL sterile 100 mg/mL SiO_2_ suspension (SiO_2_ and BIC groups) or NS (control group), respectively. At 4 weeks after instillation, rats received either 0.5% CMC-Na or 200 mg/kg BIC via intragastric administration once a day. At 8 weeks after BIC treatment, rats were anesthetized and tissues extracted for analysis as described in Supplementary Materials and Methods.

### Cell culture

RAW264.7, NIH-3T3, and TC-1 cells were obtained from American Type Culture Collection (ATCC, Manassas, VA, USA) and maintained according to the manufacturer’s recommendations. RAW264.7 and NIH-3T3 cell lines were cultured in DMEM and TC-1 cells in RPMI-1640 medium. All cells were cultured at 37 °C and 5% CO_2_ in media supplemented with 10% fetal bovine serum (FBS) and 1% penicillin and streptomycin.

### Pulmonary function tests (PFTs)

Comprehensive and reproducible pulmonary function tests (PFTs) were performed using a FlexiVent instrument with a forced oscillation system (SCIREQ, Montreal, Quebec, Canada) according to the manufacturer’s protocol. Static compliance (Cst) and inspiratory capacity (IC) were obtained at the maximum PV cycling maneuver. Negative pressure forced expiration (NPFE) attempted to mimic clinical spirometry to obtain the forced vital capacity (FVC), time to peak expiratory flow (TPEF), forced expiratory volume at x second (FEVx), and forced expiratory flow at x second (FEFx) values. The Snap shot-90 maneuver was used to measure resistance (Rrs), compliance (Crs), and elastance (Ers) of the whole respiratory system. Newtonian resistance (Rn), tissue damping (G), tissue elastance (H), and tissue hysteresivity (G/H) values were obtained using the Quick Prime-3 system. All maneuvers were performed until three stable values were recorded.

### Bronchoalveolar lavage fluid (BALF) collection

Following anesthesia, the right lung of rat was separated and ligated, followed by bronchoalveolar lavage in the left lung using cold NS three times. BALF was collected and subsequently centrifuged at 1000 r/min for 15 min at 4 °C. Cell deposits were resuspended in cold NS for further use.

### Statistical analysis

All statistical tests were performed using Prism 7.0 (GraphPad Software, La Jolla, USA). For all the graphs, data are presented as mean ± S.E.M. Differences were considered statistically significant at *P* < 0.05. Comparisons between multiple groups were conducted using two-way ANOVA followed by Tukey post hoc-test.

## Results

### BIC improves pulmonary function of silicosis model rats

To assess the efficacy of BIC against silicosis, we established an in vivo model via intratracheal instillation of a SiO_2_ suspension in rats (Figure S1A). At 4 weeks after instillation, abundant silicotic nodules and aberrant collagen deposition were observed in lungs of exposed rats, accompanied by marked loss of body weight, compared with the group receiving NS only (Figure S1B-C), suggestive of significant health damage during the development of silicosis. Slight recovery of body weight was observed in silicosis-induced rats receiving BIC intragastrically for 8 weeks, indicating potential efficacy of BIC against silicosis (Figure S1D-E). The following PFTs, including spirometry (FVC, FEVx, TPEF, and FEFx), Rrs, Ers, Crs, Rn, G, H, stiffness degree (Cst), and IC, were performed for further assessment of the activity of BIC against silicosis. Notably, the majority of PFT indicator values deteriorated in rats with progressive silicosis, which were recovered to varying extents following BIC treatment (Table 1). Among the PFT indicators examined, BIC induced a significant increase in the FEV0.2/FVC ratio and FEF0.1 and decrease in TPEF compared with the corresponding values in SiO_2_-treated rats, indicating that BIC enhances the expiratory capacity in rats with silicosis. Additionally, BIC treatment led to an increase in IC, an indicator of inspiratory capacity, and improved Cst, a marker of pulmonary stiffness, and pressure-volume loops (PV loops) of rats exposed to silica (Figure S1F) [19]. The effects of BIC on these indicators clearly supports enhancement of lung function in rats with silicosis.

**Table 1:** Overview of all pulmonary function parameters measured using the FlexiVent instrument

Physiological significance	PFTs	Control	SiO_2_	BIC
Expiratory capacity	FEV0.05/FVC (%)	22.74 ± 1.94***	8.47 ± 0.41	8.66 ± 0.38
	FEV0.1/FVC (%)	61.81 ± 2.88***	18.87 ± 0.90	23.10 ± 1.33
	FEV0.2/FVC (%)	90.99 ± 1.37***	54.12 ± 1.67	66.15 ± 2.83***
	FEF0.05 (mL/s)	77.98 ± 9.863***	22.30 ± 0.4621	23.67 ± 0.6889
	FEF0.1 (mL/s)	61.65 ± 11.19**	24.66 ± 0.4975	56.16 ± 8.570*
	FEF0.2 (mL/s)	8.616 ± 1.419***	41.29 ± 4.464	36.50 ± 6.275
	TPEF (s)	0.04167 ± 0.002991***	0.1915 ± 0.003058	0.1401 ± 0.007048***
Inspiratory capacity	IC (mL)	12.99 ± 0.3719***	8.127 ± 0.2928	9.764 ± 0.2988**
Static compliance	Cst (mL/cmH_2_O)	1.411 ± 0.06125***	0.5847 ± 0.05721	0.8768 ± 0.04969**
Elastance	Ers (cmH_2_O/mL)	1.095 ± 0.04837***	2.577 ± 0.3897	1.951 ± 0.1893
Compliance	Crs (cmH_2_O/mL)	0.9291 ± 0.03941***	0.4447 ± 0.04983	0.5548 ± 0.05581
Resistance	Rrs (cmH_2_O.s/mL)	0.1350 ± 0.01884	0.1571 ± 0.02701	0.1337 ± 0.01876
Central airway resistance	Rn (cmH_2_O.s/mL)	0.03439 ± 0.003290***	0.07001 ± 0.006044	0.06728 ± 0.007056
Tissue damping	G (cmH_2_O/mL)	0.2930 ± 0.02932	0.4328 ± 0.04720	0.5304 ± 0.06135
Tissue elastance	H (cmH_2_O/mL)	1.071 ± 0.1216***	1.983 ± 0.1492	1.795 ± 0.1361
Tissue hysteresivity	G/H	0.2772 ± 0.008842	0.2360 ± 0.04549	0.3060 ± 0.04131

All data are presented as mean ± SEM (*p < 0.05, **p < 0.01, and ***p < 0.001 compared to the SiO_2_ group (*n* = 9 per group))

### BIC reduces inflammation and fibrosis in lungs of silicosis model rats

**Fig. 1:**
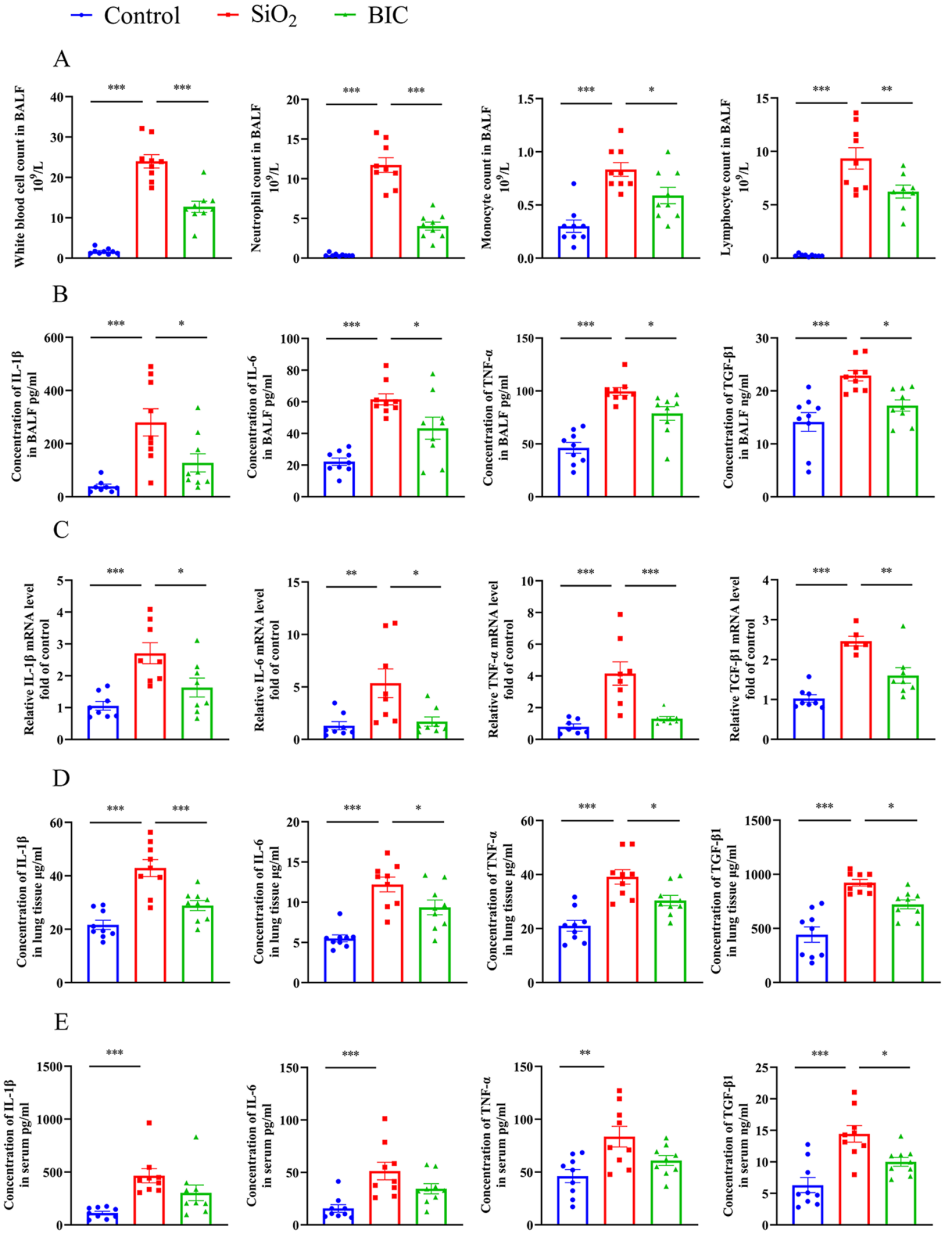
BIC reduces inflammatory cytokine secretion in a silicosis rat model. BALF from the left lung of each rat was centrifuged, and the cell precipitate and supernatant fractions collected. (**A**) Amounts of white blood cells, neutrophils, monocytes, and lymphocytes in precipitates of BALF. (**B**) ELISA of IL-1β, IL-6, TNF-α, and TGF-β1 in supernatant fractions of BALF. **C-D.** In tissues isolated from the right lung of each rat, expression of the four cytokines at the mRNA level in tissue extracts (**C**) and protein level in tissue homogenates (**D**) were analyzed. Left to right: IL-1β, IL-6, TNF-α, and TGF-β1. **E.** Serum concentrations of IL-1β, IL-6, TNF-α, and TGF-β1. Data are presented as mean ± SEM. All graphs share common symbols (blue for the Control group receiving NS intratracheally, red for the SiO_2_ treatment group, and green for the BIC group treated with SiO_2_ and receiving BIC treatment). *p < 0.05, **p < 0.01, and ***p < 0.001 compared to the SiO_2_ group. *n* = 9 per group

**Fig. 2:**
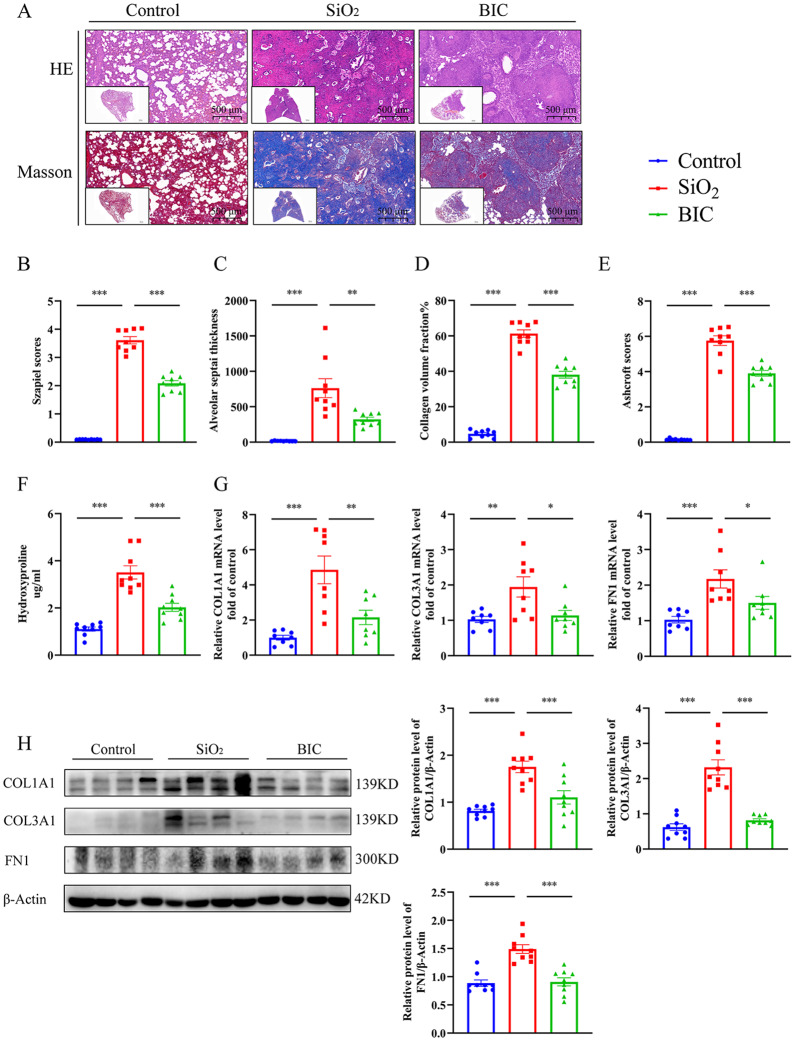
BIC alleviates inflammation and fibrosis in lungs of SiO_**2**_-induced rats. **A**. Representative photographs of lung sections from the three treatment groups of the silicosis rat model stained with either HE (above) or Masson (below) at 1× and 10× magnification. **B-C**. Assessment of inflammation in HE-stained sections of the silicosis rat model based on Szapiel Scores (**B**) and Alveolar Septal Thickness (**C**). **D-E**. Fibrosis assessment based on CVF (**D**) and Ashcroft Scores (**E**) of Masson staining in silicosis rats. **F**. Analysis of hydroxyproline in lung tissues of silicosis rats. **G**. Expression of ECM components (COL1A1, COL3A1, FN1) at the mRNA level in lung tissue extracts of the silicosis rat model. **H**. Representative images of Western blots (left) of COL1A1, COL3A1, and FN1 in lung tissue, and quantitative protein expression in lung tissue after standardization (right). All data are presented as mean ± SEM. Graphs in **B-H** share common symbols (blue for Control, red for SiO_2_, and green for BIC). *p < 0.05, **p < 0.01, and ***p < 0.001 compared to the SiO_2_ group. *n* = 8–9 per group

Inflammation induced by SiO_2_ is one of the critical triggers of silica-induced lung injury [6]. In our silicosis rat model, BIC induced a significant decrease in the extremely high levels of white blood cells, lymphocytes, neutrophils, and monocytes/macrophages in BALF of rats exposed to silica (Fig. 1A). Moreover, the expression of silicosis related cytokines (IL-1β, IL-6, TNF-α, and TGF-β1) in BALF was further detected using ELISA. BIC effectively suppressed the levels of all four cytokines in BALF (Fig. 1B). Consistently, BIC treatment led to a significant decrease in the four cytokines at both mRNA and protein levels in lung tissues of rats exposed to silica (Fig. 1C-D). While all four cytokines were upregulated in serum of rats exposed to silica, indicating a systemic inflammatory response, only TGF-β1 was suppressed to a significant extent by BIC (Fig. 1E). HE staining additionally revealed that BIC alleviated the massive immune cell infiltration and thickening of alveolar septa in lungs of silicosis model rats. However, BIC had no restorative effect on damaged alveolar structures (Fig. 2A-C), indicating activity in attenuating the degree of lung injury caused by inflammation in silicosis, rather than repair of injured tissue.

Fibrosis is another key feature of silicosis. Normally, limited collagen fiber deposition is observed in alveoli. However, following exposure to silica, abundant collagen fibers are deposited (Fig. 2A). BIC not only visually reduced aberrant collage fiber deposition, as observed from Masson staining of lung tissue, but also markedly decreased the collagen volume fraction (CFV%) and Ashcroft scores (Fig. 2A, D-E). Concomitantly, BIC treatment diminished the HYP level in lung tissues of silicosis rats (Fig. 2F). To further establish the effects of BIC on ECM production, we analyzed mRNA and protein expression of ECM components (COL1A1, COL3A1, FN1) in lung tissues. BIC significantly inhibited the synthesis of ECM components compared to the SiO_2_ group in the silicosis model (Fig. 2G-H). The collective findings clearly demonstrate that BIC suppresses the progression of silicosis through reducing the number of inflammatory cells and secretion of inflammatory cytokines as well as the production and deposition of ECM in the rat model.

### BIC regulates polarization and apoptosis of macrophages

**Fig. 3:**
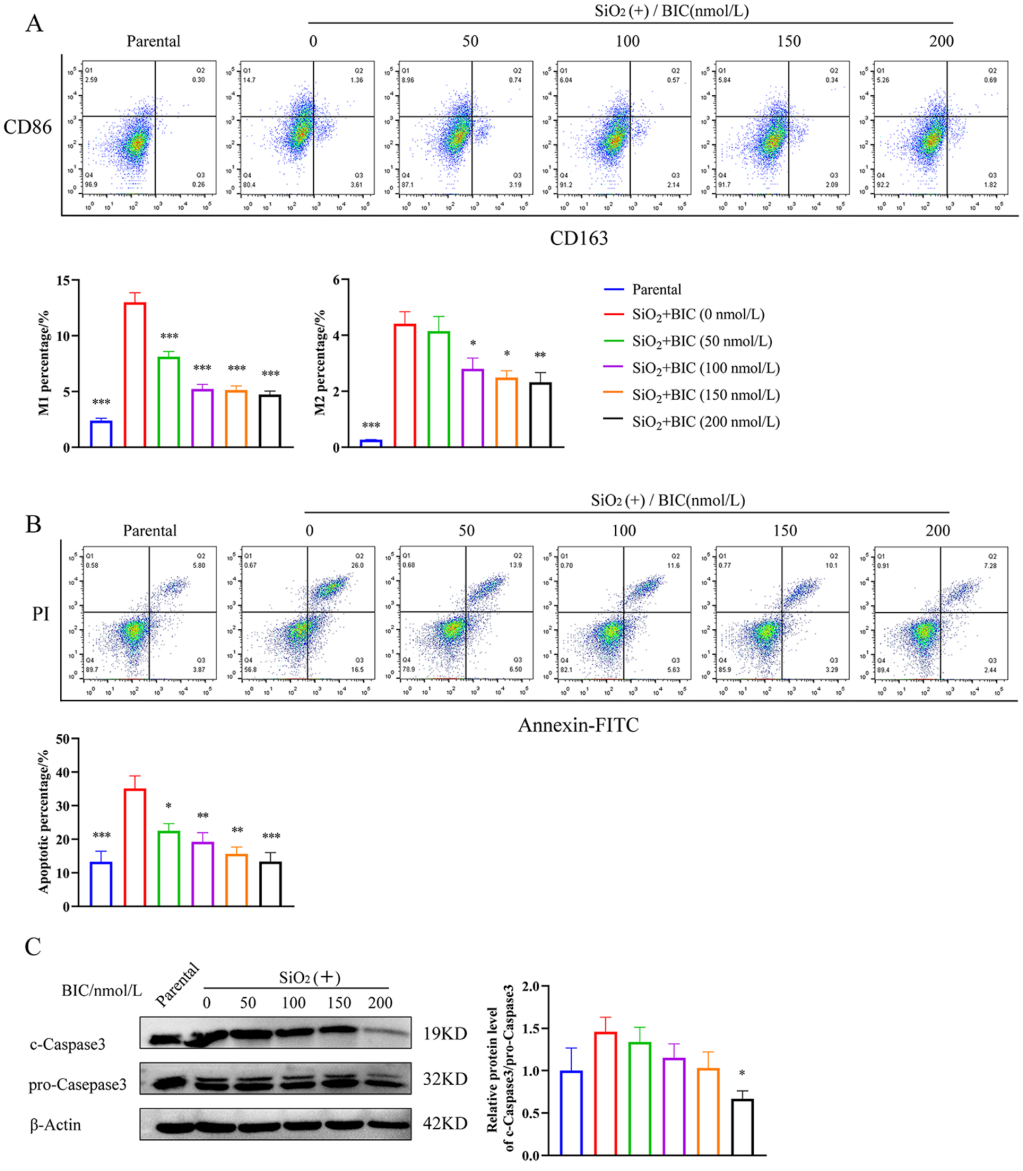
BIC suppresses SiO_2_-induced macrophage polarization, proliferation and apoptosis. RAW264.7 cells were pretreated with SiO_2_ (100 µg/mL) for 8 h, followed by treatment with different concentrations of BIC (0, 50, 100,150, and 200 nmol/L) for 48 h. The parental group was not stimulated with either SiO_2_ or BIC. **A**. Flow cytometry analysis (up) and statistical ratios of M1 and M2 polarized macrophages (down) in SiO_2_-stimulated RAW264.7 cells treated with different concentrations of BIC. **B**. Apoptosis (up) and statistical analysis (down) of the apoptotic percentage of SiO_2_-stimulated RAW264.7 cells detected via flow cytometry, using Annexin V-FITC/PI staining. **C**. Western blot analysis of the expression of apoptosis-related proteins in SiO_2_-stimulated RAW264.7 cells treated with different concentrations of BIC. β-Actin was used as a loading control. All data are presented as mean ± SEM. Histograms in this figure share common symbols (blue for parental, red for 0 nmol/L, green for 50 nmol/L, purple for 100 nmol/L, orange for 150 nmol/L, and black for 200 nmol/L BIC treatment after SiO_2_ stimulation). *p < 0.05, **p < 0.01, and ***p < 0.001 compared to the 0 nmol/L group

Macrophages play a critical role in silicosis. A previous study by our group on a rat silicosis model showed that BIC suppresses the aggregation and activation of macrophages through down-regulation of Arg1 and iNOS [18]. Consistently, BIC induced down-regulation of Arg1 and iNOS induced by SiO_2_ both in vitro and in vivo (Figure S2A-B). To further validate the effect of BIC on macrophage polarization, two cell surface markers (CD86 for M1 and CD163 for M2) were used to examine the M1 or M2 macrophage ratios in SiO_2_-stimulated RAW264.7 via flow cytometry. BIC treatment led to a dose-dependent decrease in the ratios of CD86^+^ and CD163^+^ cells in RAW264.7 macrophages activated with SiO_2_ (Fig. 3A), indicating inhibitory effects on macrophage polarization to both M1 and M2 types.

On the other hand, we observed that BIC protected RAW264.7 cells from apoptosis induced by SiO_2_ in a dose-dependent manner (Fig. 3B), accompanied by diminished expression of cleaved caspase-3 (c-caspase-3) in these cells (Fig. 3C). In addition, BIC reduced the number of TUNEL-positive cells either in damaged alveolar regions or around silicon nodules (Figure S2C), that appeared largely in lungs of rats exposed to silica, indicating a suppressive effect on macrophage apoptosis, both in vitro and in vivo.

### BIC suppresses the secretion of inflammatory cytokines in macrophages via the JAK2/STAT3/SOCS3 pathway

**Fig. 4:**
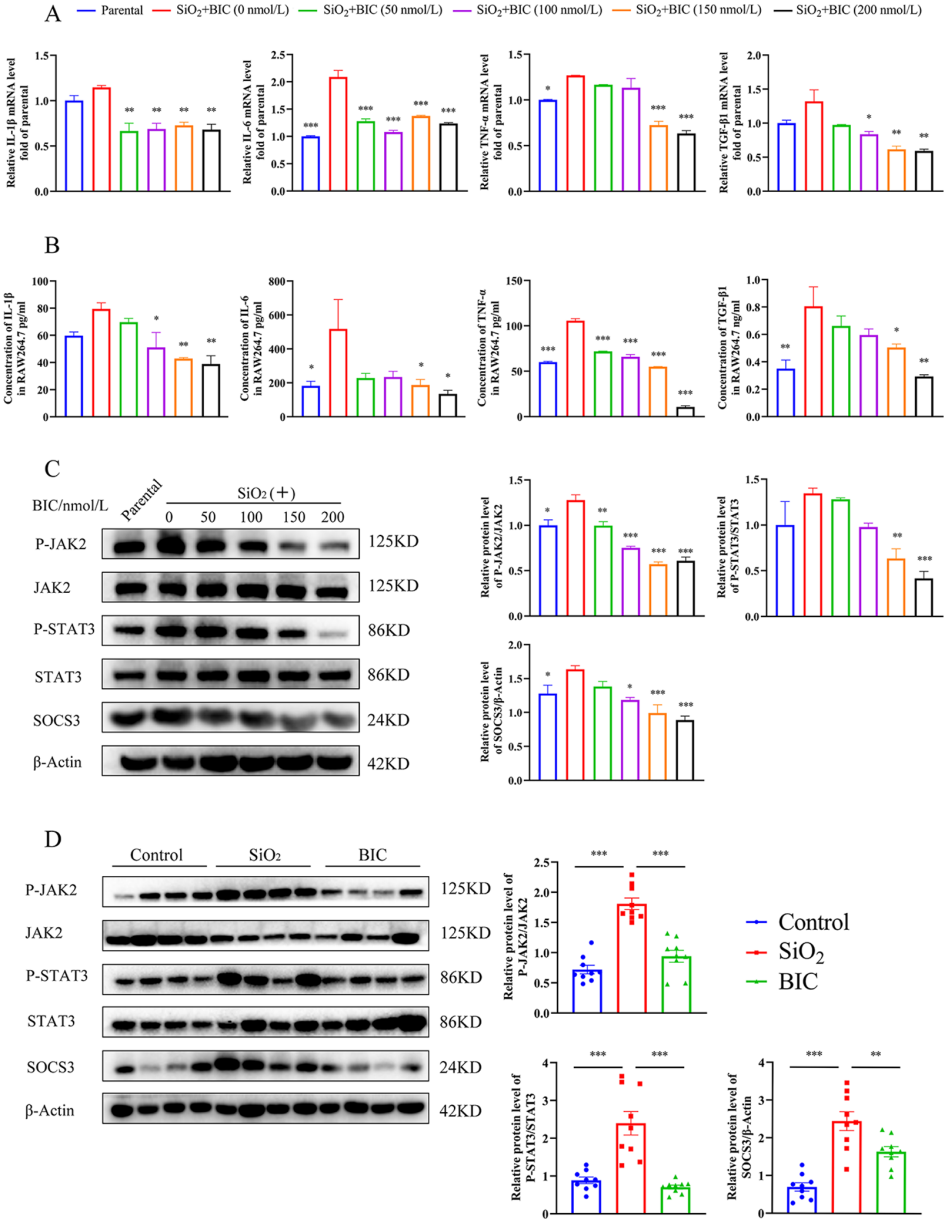
BIC suppresses inflammatory cytokine secretion in SiO_2_-induced RAW264.7 cells via the JAK2/STAT3 signaling pathway. RAW264.7 cells were pre-stimulated with the SiO_2_ suspension for 8 h, followed by treatment with different concentrations of BIC (0, 50, 100,150, and 200 nmol/L) for 48 h. The parental group was not cultured with the SiO_2_ suspension or BIC. **A**. Assessment of the four inflammatory cytokines at the mRNA level in RAW264.7 cells. Left to right: IL-1β, IL-6, TNF-α, and TGF-β1. **B**. The supernatant of RAW264.7 cells was collected after treatment with BIC and ELISA performed for analysis of inflammatory cytokines secreted into the medium. Left to right: IL-1β, IL-6, TNF-α, and TGF-β1. **C**. Analysis of activation of JAK2/STAT3 signaling based on the expression of SOCS3 and phosphorylation levels of both JAK2 and STAT3 in SiO_2_-stimulated RAW264.7 cells using western blot assay (left). The density scanning was also performed (right). **D**. Western blot analysis (left) and the density scanning (right) of JAK2/STAT3 signaling in lung tissue extracts of the silicosis rat model (*n* = 9). All data are presented as mean ± SEM. Graphs in **B** and **C** share common symbols with those in **A** (blue for parental, red for 0 nmol/L, green for 50 nmol/L, purple for 100 nmol/L, orange for 150 nmol/L, and black for 200 nmol/L BIC treatment after SiO_2_ stimulation). *p < 0.05, **p < 0.01, and ***p < 0.001 compared to the 0 nmol/L group in **A-C** or to the SiO_2_ group in **D**

Further, we examined whether the BIC-mediated reduction of cytokine secretion could be induced through inhibition of macrophage polarization. To this end, expression of the above four cytokines was examined in SiO_2_-stimulated RAW264.7 cells and supernatant fractions. As expected, mRNA expression of all four cytokines was suppressed and their secreted concentrations into the supernatant decreased by BIC in a dose-dependent manner (Fig. 4A-B).

The JAK2/STAT3 pathway is proposed to play a key regulatory role in the production of inflammatory cytokines [20]. Accordingly, we examined the phosphorylation levels of both JAK2 and STAT3 in RAW264.7 cells stimulated with SiO_2_. BIC inhibited both JAK2 and STAT3 phosphorylation in these cells in a dose-dependent manner, which was accompanied by downregulation of SOCS3, a key factor downstream of JAK2/STAT3 signaling that plays a regulatory role in cytokine transcription (Fig. 4C). Consistently, abnormally activated JAK2 and STAT3 as well as aberrantly expressed SOCS3 in lung tissues of silicosis rats were downregulated by BIC in the silicosis model (Fig. 4D and Figure S3). Our findings clearly support the theory that BIC reduces inflammation in silicosis via inhibitory effects on the JAK2/STAT3/SOCS3 signaling pathway.

### BIC inhibits EMT of pulmonary epithelial cells via suppressing the TGF-β1/SMAD2/3 signaling pathway

**Fig. 5:**
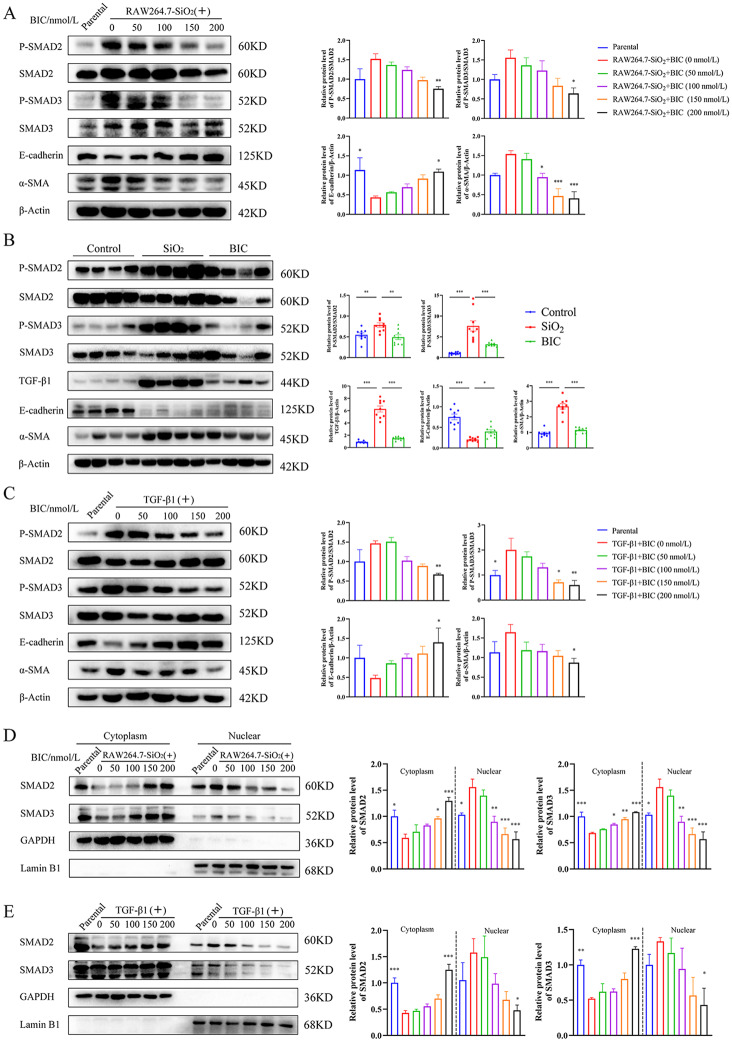
BIC inhibits EMT through suppression of both phosphorylation and translocation of SMAD2 and SMAD3. **A**. Mouse lung epithelial TC-1 cells were pre-treated with supernatant of SiO_2_-stimulated RAW264.7 cells for 48 h, followed by treatment with different concentrations of BIC (0, 50, 100, 150, and 200 nmol/L) for 48 h. Cells in the parental group were co-cultured with supernatant of non-treated RAW264.7 cells. Western blot (left) and the relative quantitative analysis (right) were performed to establish the expression of EMT markers as well as TGF-β1/SMAD signaling. **B**. Detection of EMT markers and molecules of the TGF-β1/SMAD signaling pathway in lung tissue extracts of the silicosis rat model, using western blot assay. β-Actin was used as a loading control (*n* = 9). **C**. TC-1 cells were stimulated with TGF-β1 (10 ng/mL) for 48 h and subsequently treated with different concentrations of BIC (0, 50, 100, 150, and 200 nmol/L) for 48 h. The parental group was cultured with no TGF-β1 and BIC. Western blot analysis of EMT markers and components of the TGF-β1/SMAD signaling pathway. The density scanning was also performed. **D-E**. Effects of BIC on nuclear translocation of SMAD2 and SMAD3 in TC-1 cells treated with either SiO_2_-stimulated RAW264.7 cells (**D**) or TGF-β1 (**E**). GAPDH and Lamin B1 were used as loading controls for cytoplasm and nuclear, respectively. Graphs in **D** share common symbols with those in **A.** Graphs in **E** share common symbols with those in **C.** Blue for parental, red for 0 nmol/L, green for 50 nmol/L, purple for 100 nmol/L, orange for 150 nmol/L, and black for 200 nmol/L BIC treatment co-cultured with the supernatant of SiO_2_-stimulated RAW264.7 cells or TGF-β1. *p < 0.05, **p < 0.01, and ***p < 0.001 compared to the 0 nmol/L group in **A and C-E** or to the SiO_2_ group in **B**

It is reported that pulmonary epithelial cells undergo EMT for conversion to fibroblasts, contributing to the accumulation of ECM [21–23]. Interestingly, stimulation by simply adding SiO_2_ particles alone into culture medium did not obviously induce EMT in murine pulmonary epithelial TC-1 cells (Figure S4A). Accordingly, RAW264.7 macrophages were initially stimulated with SiO_2_ particles, followed by co-culture of TC-1 cells with supernatant of SiO_2_-stimulated RAW264.7 cells, and the EMT markers, E-cadherin and α-SMA, analyzed. As expected, the supernatant of SiO_2_-stimulated RAW264.7 cells induced significant up-regulation of α-SMA and down-regulation of E-cadherin in TC-1 cells, which was blocked by BIC in a dose-dependent manner (Fig. 5A). Consistently, BIC promoted E-cadherin and decreased α-SMA expression in the lung of silicosis rats relative to the SiO_2_ group (Fig. 5B and Figure S4B), indicating effective suppression of the EMT process during the development of silicosis.

To further identify the inflammatory cytokines secreted by macrophages involved in inducing EMT in pulmonary epithelial cells that are targeted by BIC, TC-1 cells were exposed to IL-1β, IL-6, TNF-α, and TGF-β1. Among these molecules, only TGF-β1 promoted EMT progression of TC-1 cells (Figure S4A). Earlier reports have demonstrated that TGF-β1 activates SMAD2/3 for binding to promoters of fibrosis genes and modulates progression of EMT [24, 25]. Increased phosphorylation of SMAD2/3 was observed in both lungs of silicosis rats and TC-1 cells cultured with the supernatant of SiO_2_-stimulated RAW264.7 cells or TGF-β1 alone, which was inhibited by BIC (Fig. 5A-C). The data suggest that BIC inhibits EMT by suppressing the activation of SMAD2/3 induced by TGF-β1.

Phosphorylated SMAD2/3 forms a complex with SMAD4 that translocates to the nucleus to activate pro-fibrotic genes and regulate EMT development [25]. Expression of SMAD2/3 in the cytoplasm and nucleus of TC-1 cells was further examined. Both SMAD2 and SMAD3 accumulated in the nucleus of TC-1 cells cultured with either the supernatant of SiO_2_-stimulated RAW264.7 cells or TGF-β1 alone. Accumulation of SMAD2/3 was suppressed by BIC in a dose-dependent manner (Fig. 5D-E), leading to the proposal that BIC blocks nuclear translocation of SMAD2 and SMAD3 from the cytoplasm. Taken together, our findings suggest that BIC inhibits the EMT process induced by TGF-β1 in pulmonary epithelial cells via effects on both dephosphorylation and nuclear translocation of SMAD2 and SMAD3.

### BIC inhibits FMT in fibroblasts in a silicosis murine in vitro model

**Fig. 6:**
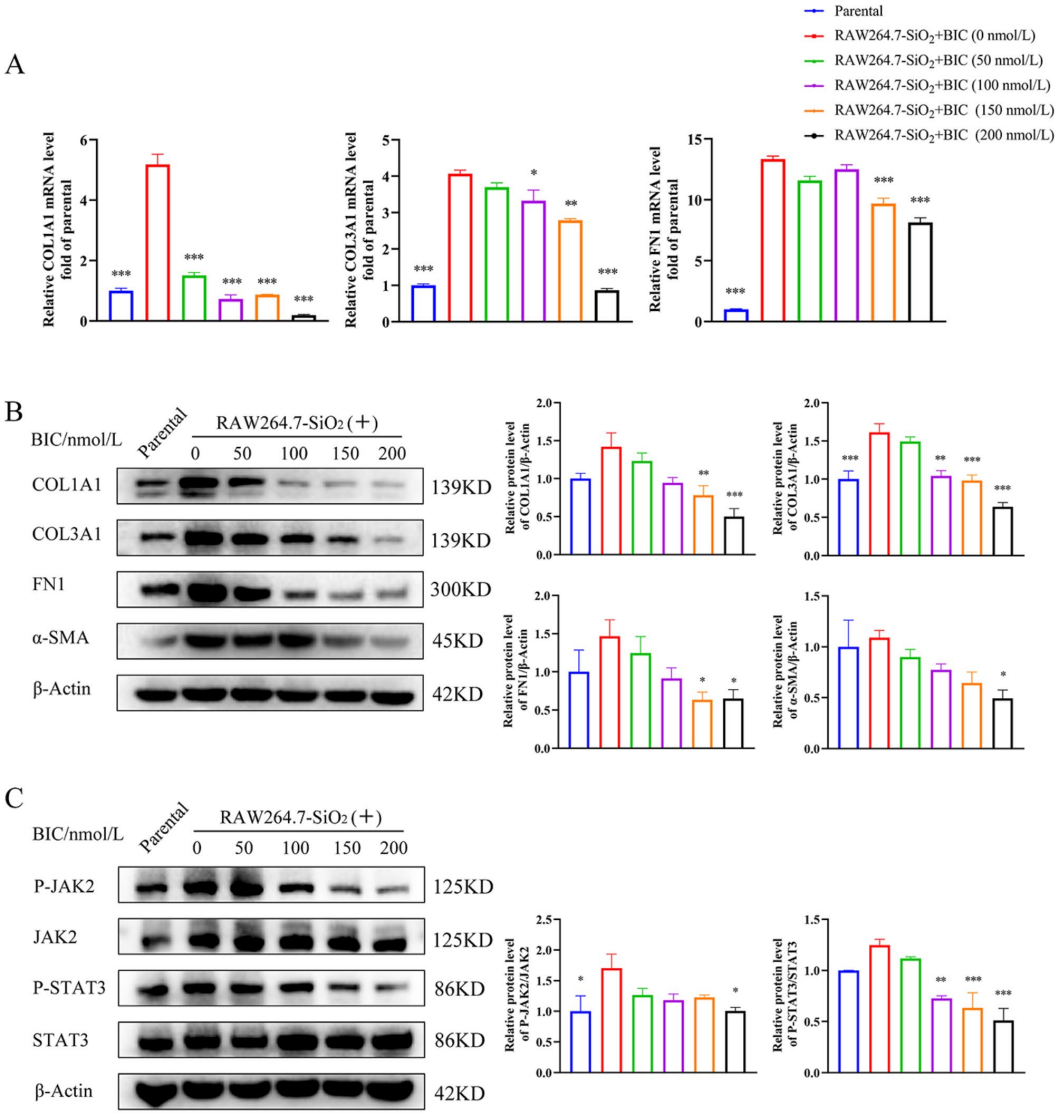
BIC inhibits FMT in fibroblasts via the JAK2/STAT3 signaling pathway. The FMT model was established by co-culturing NIH-3T3 cells with the supernatant of SiO_2_-stimulated RAW264.7 cells. Co-cultured cells were treated with different concentrations of BIC (0, 50, 100,150, and 200 nmol/L). Cells in the parental group were co-cultured with the supernatant of non-treated RAW264.7 cells. **A**. mRNA expression of ECM components synthesized by NIH-3T3 cells. Left to right: COL1A1, COL3A1, FN1. **B**. Western blot (left) and relative quantitative analysis (right) of FMT markers and ECM components in co-cultured NIH-3T3 cells. **C**. Analysis of activation of the JAK2/STAT3 pathway in NIH-3T3 cells co-cultured with RAW264.7 using western blot assay. Density scanning results are presented as well. β-Actin was used as a loading control. All data are presented as mean ± SEM. Histograms in **A, B,** and **C** share common symbols (blue for parental, red for 0 nmol/L, green for 50 nmol/L, purple for 100 nmol/L, orange for 150 nmol/L, and black for 200 nmol/L BIC treatment co-cultured with the supernatant of SiO_2_-stimulated RAW264.7 cells). *p < 0.05, **p < 0.01, and ***p < 0.001 compared to the 0 nmol/L group

Fibroblasts undergoing FMT is another key process in fibrotic disease [26]. To validate the effects of BIC on fibroblasts, we established an FMT model using NIH-3T3 cells cultured with the supernatant of SiO_2_-stimulated RAW264.7 cells and treated with different concentrations of BIC. Notably, BIC suppressed expression at mRNA and protein levels of both the FMT marker, α-SMA, and ECM components (COL1A1, COL3A1, and FN1) induced by the supernatant of SiO_2_-stimulated RAW264.7 cells in NIH-3T3 cells in a dose-dependent manner (Fig. 6A-B). Furthermore, BIC suppressed the phosphorylation of JAK2 and STAT3 in NIH-3T3 cells (Fig. 6C). The results indicate that BIC prevents fibroblasts from undergoing FMT induced by macrophage-secreted inflammatory cytokines via regulation of the JAK2/STAT3 signaling pathway.

**Fig. 7:**
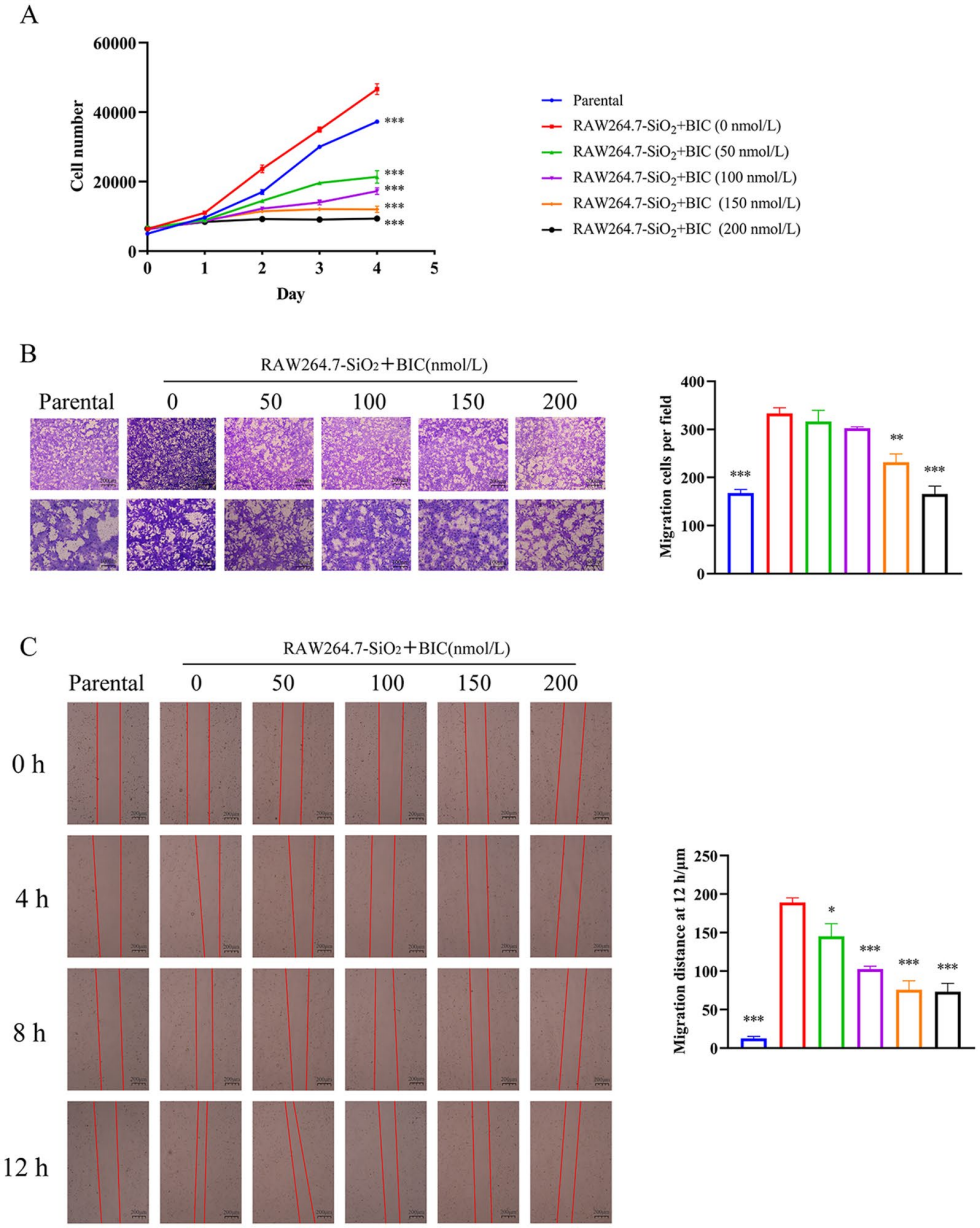
BIC suppresses fibroblasts proliferation, migration, and invasion in vitro. **A**. NIH-3T3 cells were seeded into 96-well plates with the supernatant of SiO_2_-stimulated RAW264.7 cells for 12 h and then treated with different concentrations of BIC (0, 50, 100,150, and 200 nmol/L) for 0–4 days. Cell proliferation assay using CCK-8 was performed to evaluate the effects of BIC on proliferation these cells. **B**. The transwell migration assay was conducted in NIH-3T3 cells simultaneously treated with the supernatant of SiO_2_-treated RAW264.7 and BIC for 12 h. Representative micrographs and quantified migratory cells per field of each group are shown. Up: 4×, down: 10×. **C**. Wound healing assay of NIH-3T3 cells co-cultured with SiO_2_-treated RAW264.7. Wounds were measured at 0, 4, 8, and 12 h after BIC treatment. Representative images of each group at different time points are shown (4×). Quantitative analysis of scratch wound healing assay after 12 h are presented as well. All data are presented as mean ± SEM. The graphs in **A, B,** and **C** share common symbols (blue for parental, red for 0 nmol/L, green for 50 nmol/L, purple for 100 nmol/L, orange for 150 nmol/L, and black for 200 nmol/L BIC treatment co-cultured with the supernatant of SiO_2_-stimulated RAW264.7 cells). *p < 0.05, **p < 0.01, and ***p < 0.001 compared to the 0 nmol/L group

Earlier research suggests that fibroblasts undergo FMT for proliferation and migration and thus play an important role in tissue repair [27]. The influence of BIC on proliferation and migration of NIH-3T3 cells cultured with supernatant of SiO_2_-stimulated RAW264.7 cells was determined using cell proliferation and transwell migration assays, as described in Supplementary Materials and Methods. As expected, BIC induced significant suppression of proliferation and migration of NIH-3T3 cells induced by the supernatant of SiO_2_-stimulated RAW264.7 cells (Fig. 7A-B). Furthermore, BIC slowed wound healing in a dose-dependent manner, another process involving FMT, in NIH-3T3 cells cultured with SiO_2_-stimulated RAW264.7 cell supernatant fractions (Fig. 7C). Our collective results demonstrate that BIC ameliorates FMT through inhibiting JAK2/STAT3 signaling in fibroblasts to attenuate fibrotic tissue repair.

### BIC regulates TGF-β1 secreted by macrophages to suppress FMT in fibroblasts via the TGF-β1/JAK2/STAT3 signaling pathway

**Fig. 8:**
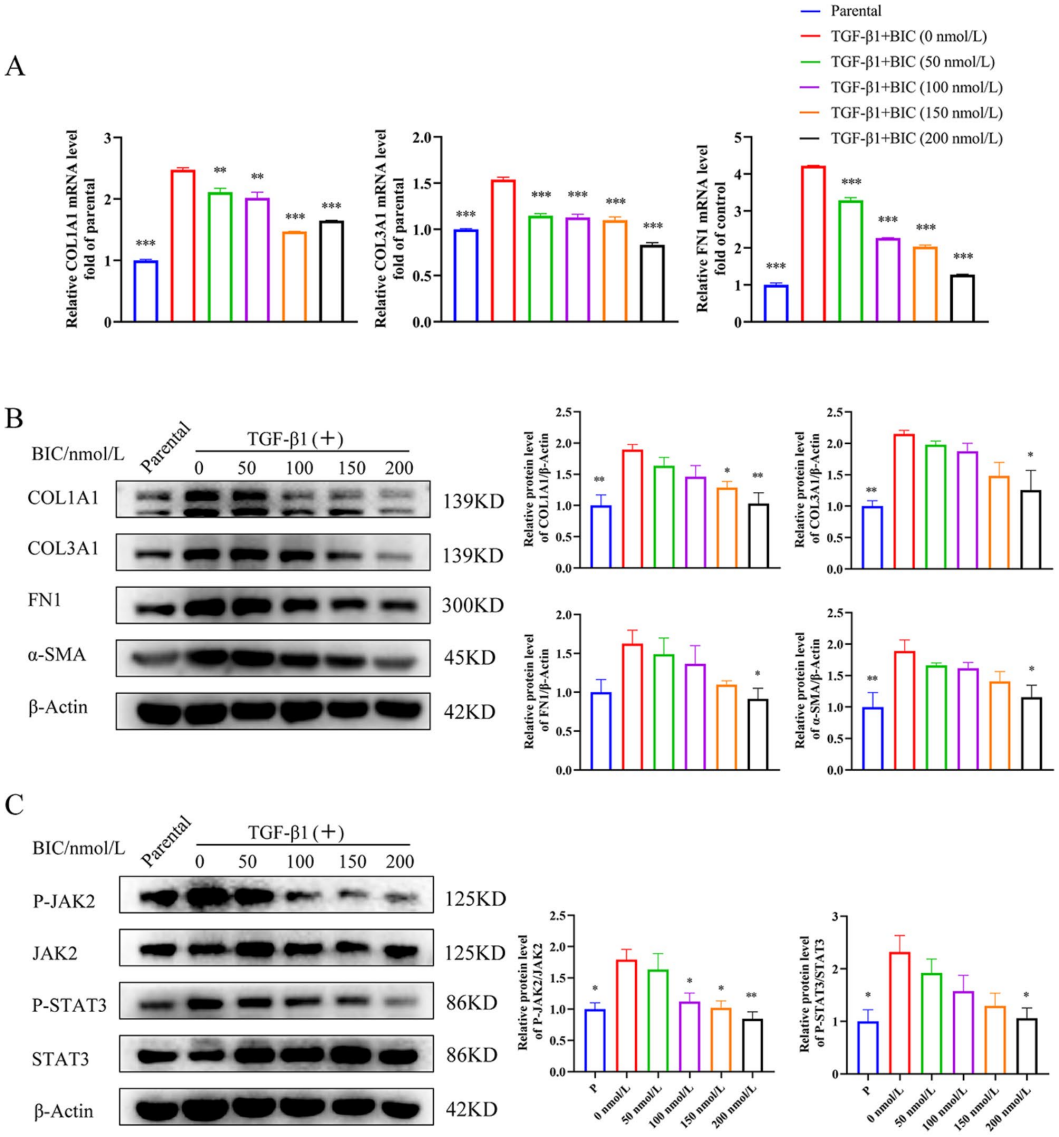
BIC suppresses TGF-β1-induced FMT of fibroblasts through the JAK2/STAT3 signaling pathway. NIH-3T3 cells were pre-stimulated with TGF-β1 (10 ng/mL) for 12 h before the addition of BIC (0, 50, 100, 150, and 200 nmol/L) into the culture medium. **A**. Relative expression of ECM components (COL1A1, COL3A1, FN1) at mRNA level using quantitative PCR assay. **B**. Western blot analysis (left) and relative density scanning (right) of ECM components and FMT markers. β-Actin was used as a loading control. **C**. Phosphorylation of JAK2 and STAT3 in TGF-β1- stimulated NIH-3T3 cells. The density scanning was presented as well. All data are presented as mean ± SEM. The histograms share common symbols (blue for parental, red for 0 nmol/L, green for 50 nmol/L, purple for 100 nmol/L, orange for 150 nmol/L, and black for 200 nmol/L BIC treatment co-cultured with TGF-β1). *p < 0.05, **p < 0.01, and ***p < 0.001 compared to the 0 nmol/L group

**Fig. 9:**
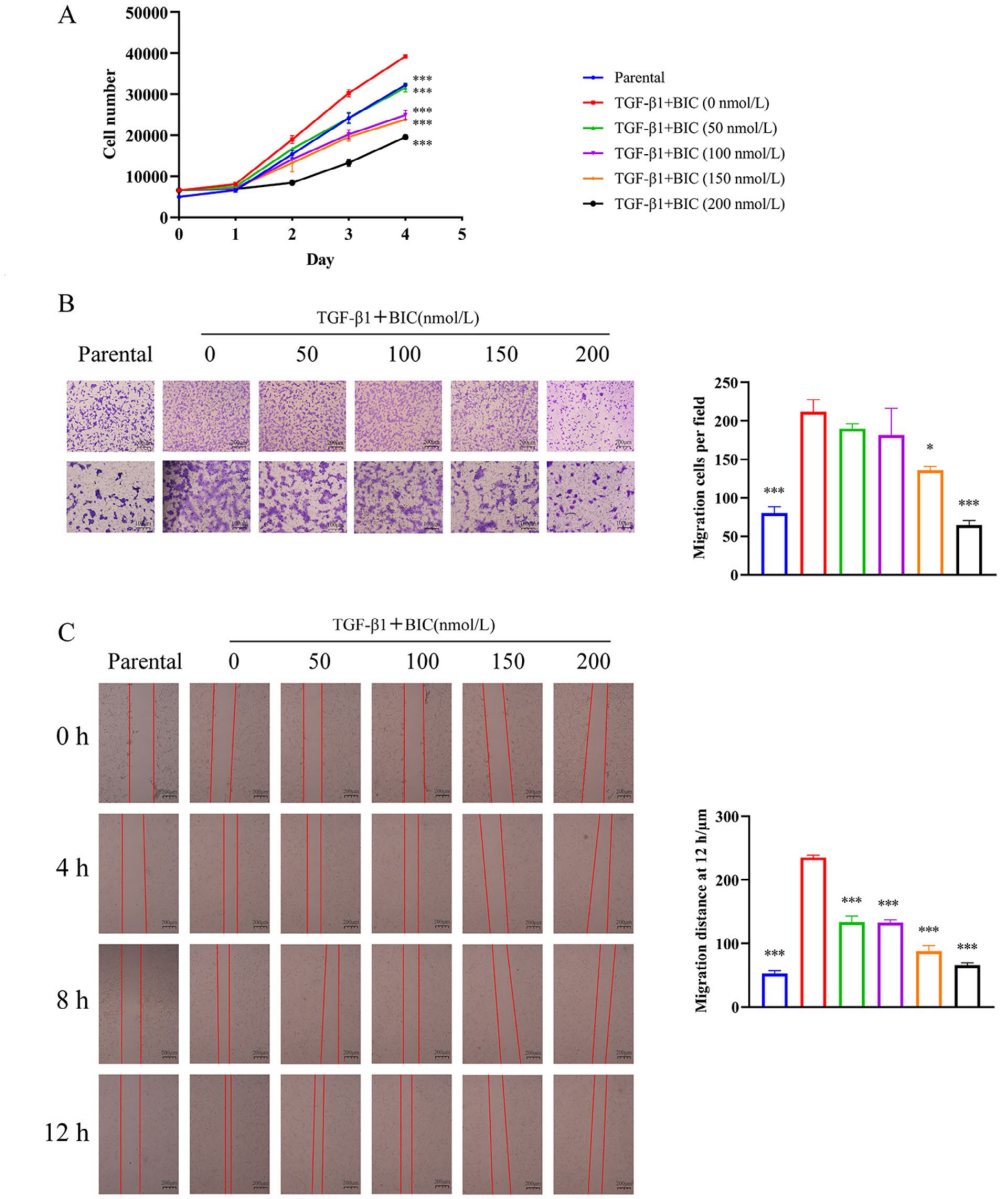
BIC suppresses TGF-β1-induced proliferation, migration, and invasion of fibroblasts through the JAK2/STAT3 signaling pathway. **A**. NIH-3T3 cells were pre-stimulated with TGF-β1 (10 ng/mL) for 12 h and added BIC (0, 50, 100, 150, and 200 nmol/L) into the culture medium for 0–4 days. Proliferation capability of TGF-β1-stimulated NIH-3T3 cells was analyzed using CCK-8 proliferation assay. **B**. NIH-3T3 cells were seeded into the upper layer of the transwell chamber and treated with TGF-β1 (10 ng/mL) and different concentrations of BIC for 12 h. Representative photographs (left) and migrated cell counts (right) are presented. Magnification: up: 4×, down: 10×. **C**. Assessment of the effect of BIC on migration in NIH-3T3 cells pre-treated with TGF-β1 using the wound healing assay. Wound gaps were photographed and measured at 0, 4, 8, and 12 h after BIC treatment. Representative images of each group at four different time-points are shown (4×). Quantitation of the wound healing assay results at 12 h are presented as well. All data are presented as mean ± SEM. Graphs in **A**, **B,** and **C** share common symbols (blue for parental, red for 0 nmol/L, green for 50 nmol/L, purple for 100 nmol/L, orange for 150 nmol/L, and black for 200 nmol/L BIC treatment co-cultured with TGF-β1). *p < 0.05, **p < 0.01, and ***p < 0.001 compared to the 0 nmol/L group

To identify the cytokines secreted by macrophages that play a major role in FMT, NIH-3T3 cells were treated with the main cytokines secreted by SiO_2_-stimulated RAW264.7 cells, specifically, IL-1β, IL-6, TNF-α, and TGF-β1, as well as SiO_2_ particles. The FMT markers COL1A1, COL3A1, FN1, and α-SMA were analyzed in these cells. As expected, TGF-β1 was the only cytokine secreted by macrophages to drive FMT in fibroblasts (Figure S5). Consistent with our previous findings (Fig. 6), BIC suppressed COL1A1, COL3A1, FN1, and α-SMA at both the mRNA and protein levels in a dose-dependent manner, which were up-regulated in TGF-β1-pretreated NIH-3T3 cells (Fig. 8A-B). Activation of JAK2 and STAT3 by either the supernatant of SiO_2_-stimulated RAW264.7 or TGF-β1 was further blocked by BIC in a dose-dependent manner (Figs. 6C and 8C). BIC additionally suppressed the proliferation, migration, and wound healing capabilities of TGF-β1-pretreated NIH-3T3 cells (Fig. 9A-C), similar to the data obtained with NIH-3T3 cells co-cultured with supernatant, suggesting that TGF-β1 secreted by macrophages may serve as a key factor in BIC-mediated suppression of FMT during silicosis. Our collective results suggest that BIC inhibits fibroblast activation to attenuate fibrosis via the TGF-β1/JAK2/STAT3 signaling pathway.

## Discussion

Silicosis is a progressive, irreversible, and often fatal occupational disease caused by the inhalation, deposition, and retention of silica particles. The pathogenesis of silicosis has not been fully elucidated and therapeutic interventions are still limited due to inadequate basic knowledge [2]. Few effective drugs for silicosis are currently in clinical development. BIC derived from the traditional Chinese medicine (TCM) *Schisandra chinensis* (Wuweizi) of North, is an approved hepatoprotective drug with well-characterized safety [11]. Recent studies on different fibrotic animal models for example, bile duct ligation or dimethylnitrosamine-induced liver fibrosis [28, 29], renal interstitial fibrosis induced by unilateral ureteral obstruction [17], and bleomycin-induced idiopathic pulmonary fibrosis (IPF) [30] have demonstrated the efficacy of BIC against fibrosis. As expected, BIC exerted a significant therapeutic effect on SiO_2_-induced pulmonary fibrosis in the aspects of improvement of pulmonary function, attenuation of inflammation, and slowing down progression of fibrosis in the current study, supporting its efficacy as a potent therapeutic agent for silicosis.

Impairment of pulmonary function increases with progression of silicosis, even after the patient is no longer exposed to silica [31]. In the early stages of silicosis, patients may display no abnormalities in pulmonary function. With disease progression, architectural distortion leads to deterioration of pulmonary function [32]. Silicosis patients with poor pulmonary function have a lower quality of life and higher risk of mortality than the patient groups with better pulmonary function [33]. Thus, PFTs were employed as a means to assess the efficacy of BIC in addition to pathological staining. Items indicating lung compliance, such as Cst, or expiratory capacity, such as FEV/FVC, FEF, and TPEF, were abnormal in the SiO_2_ group, compared with the control group, suggestive of reduced pulmonary function in silicosis rats [19]. All the parameters were recovered to varying extents in silicosis rats after BIC treatment, associated with reduction of fibrosis in lungs, as detected with pathological staining. While abnormalities of pulmonary function parameters in silicosis could be either obstructive, restrictive or mixed and not specific for assessment of the therapeutic effects of BIC, improvements in pulmonary function could still be effectively used as an indicator of the efficacy of BIC.

Inflammation is considered the first stage of silicosis. Silica particles enter the lungs through the respiratory tract, where they continuously stimulate AMs to release substantial amounts of inflammatory factors (IL-1β) and induce apoptosis [34]. Damaged AMs and particles that are not eliminated in a timely manner accumulate and provoke an immune response, which triggers proliferation of neutrophils, macrophages, and lymphocytes, as well as generation of TGF-β1, IL-1β, TNF-α, and IL-6 [6]. Among them, IL-1β, IL-6, TNF-α, and TGF-β1 have been identified as key pro-inflammatory and pro-fibrotic cytokines in silicosis [6]. These cells and cytokines further aggravate the inflammatory response and promote fibrotic progression. Hence, it is possible to delay silicosis development through suppression of inflammation. BIC, an anti-inflammatory drug, protects the liver by inhibiting inflammation [35, 36]. Unsurprisingly, our data showed that BIC exerted anti-inflammatory effects in response to silica particles in many respects, such as, reducing infiltration of inflammatory cells including lymphocytes, neutrophils, and monocytes/macrophages, diminishing the secretion of inflammatory cytokines.

Macrophages are necessary for lung protection against silica-induced damage. However, aberrant aggregation and activation as well as M1/M2 polarization of macrophages may further aggravate silicosis [37]. M1 macrophages produce the pro-inflammatory cytokines IL-1β, IL-6, and TNF-α that induce inflammation while M2 macrophages synthesize the fibrotic cytokine TGF-β1 that plays a role in tissue repair [38, 39]. In our study, BIC protected macrophages against polarization and apoptosis and reduced inflammatory cell aggregation and infiltration into lungs. On the other hand, inhibition of both M1 and M2 polarization in macrophages by BIC led to decreased secretion of the four cytokines, IL-1β, IL-6, TGF-β1, and TNF-α, which contribute to amplification of the inflammation reaction and activation of myofibroblasts. Taken tighter, suppression of inflammation by BIC ultimately slowed down fibrosis progression.

TGF-β1 has been identified as a key inflammatory cytokine. The predominant role of TGF-β1 in silicosis is the promotion of fibroblast proliferation, myofibroblast differentiation, and collagen synthesis through autocrine and paracrine mechanisms [40]. BIC is reported to inhibit the expression of TGF-β1 in liver to reduce fibrosis [29, 41]. Coincident with earlier findings, BIC suppressed TGF-β1 expression in BALF, serum, and lung tissues in our silicosis rat model, while the three other cytokines examined (IL-1β, IL-6, and TNF-α) were only decreased in BALF and lung tissue and not in serum. Moreover, TGF-β1 was the only inflammatory cytokine that induced both EMT and FMT processes in vitro, similar to that observed with the supernatant of SiO_2_-stimulated RAW264.7 cells. Notably, in our study, TGF-β1 failed to stimulate inactive macrophages directly as observed with SiO_2_ particles, indicating that TGF-β1 promotes pulmonary fibrosis induced by SiO_2_ rather than initiating inflammatory progression. Thus, other initiation mechanisms that are stimulated by SiO_2_ to induce macrophage production of TGF-β1 may exist. Importantly, BIC could block both EMT and FMT processes induced by either direct addition of TGF-β1 or the supernatant containing TGF-β1 in a dose-dependent manner, suggesting that TGF-β1 could serve as a therapeutic target of BIC against silicosis.

TGF-β1 promotes phosphorylation of the signal transducer proteins SMAD2/3, following which phosphorylated SMAD2/3 forms a heterotrimeric complex with SMAD4 and translocates into the nucleus. The complex binds a consensus sequence and regulates fibrotic gene transcription, which is described as a canonical (SMAD-dependent) pathway [25]. Activation of canonical signaling induces loss of phenotype of epithelial cells and EMT, which is characterized by low expression of epithelial markers (E-cadherin) and high expression of mesenchymal markers (α-SMA) [42], leading to significantly increased production of ECM components [43]. In our experiments, BIC blocked phosphorylation or nuclear translocation of SMAD2/3 in TC-1 cells co-cultured with SiO_2_-stimulated macrophages or TGF-β1, indicating that inhibition of the canonical TGF-β1 signaling pathway in lung epithelial cells to suppress EMT may be a key strategy of BIC to reduce fibrosis.

In addition to the canonical signaling pathway, TGF-β1 activates a variety of SMAD-independent pathways (known as non-canonical signaling) to modify cell function. These non-SMAD pathways include MAPK, PI3K/AKT, and Rho-like GTPase signaling [8, 44]. In fibroblasts and fibrotic diseases, such as systemic sclerosis and CCl_4_-induced liver fibrosis, another SMAD-independent TGF-β1 activation pathway has been described that activates JAK2 and STAT3 [45–48]. Abnormalities of the JAK/STAT signaling pathway play important roles in both cancer progression and inflammatory and autoimmune diseases [49–51]. Inhibition of both p-JAK2 and p-STAT3 protected lung fibroblasts from undergoing FMT in IPF [45, 52]. Similarly, in our silicosis model, both JAK2 and STAT3 were activated by TGF-β1 in fibroblasts, but not the other three cytokines, leading to promotion of the FMT process and wound healing. Our findings indicate that FMT in fibroblasts driven by JAK2/STAT3 signaling could present another pathway for accumulation of ECM. Consistently, upon BIC-mediated down-regulation of phosphorylated JAK2 and STAT3 in silicosis rat lung and NIH-3T3 cells via suppression of TGF-β1 secretion, ECM deposition was markedly decreased in both tissues and cultured cells, indicating that fibroblast activation and FMT via TGF-β1/JAK2/STAT3 signaling may serve as another mechanism by which BIC targets TGF-β1 to exert therapeutic effects against silicosis.

Other than driving FMT, the JAK2/STAT3 signaling pathway is significantly activated under conditions of interactions of a range of profibrotic/pro-inflammatory cytokines during physiological and pathological processes to regulate the inflammatory response [20, 45, 53]. In our study, significant BIC-induced inhibition of the phosphorylation levels of both JAK2 and STAT3 was observed in lung tissues of the silicosis rat model and macrophages in vitro, leading to down-regulation of SOCS3 in a cascade of diminished secretion of inflammatory cytokines, such as TNF-α, IL-1β, and IL-6 [32, 33], as well as negative feedback regulation of JAK2 phosphorylation [31]. However, single cytokines, including TGF-β1, failed to activate this signaling pathway in non-stimulated macrophages as observed in fibroblasts in vitro, indicating that activation of JAK2/STAT3 signaling in macrophages presents a means to amplify rather than initiate the inflammatory response. Hence, inactivation of JAK2/STAT3 signaling in macrophages may not be the only mechanism by which BIC reduces inflammation during silicosis.

Two types of cellular transformation processes, EMT and FMT, are characteristic of fibrotic diseases, including silicosis. The two main components of lung tissues, epithelial cells and fibroblasts, undergo EMT and FMT, respectively, in response to inflammatory stimulation by silica particles at the fibrosis stage of silicosis. Consequently, alveolar epithelial cells lose their epithelial phenotype. Meanwhile, fibroblasts activate and proliferate abnormally, and subsequently lose their differentiation ability and acquire the mesenchymal phenotype of myofibroblasts, ultimately exacerbating the synthesis and abnormal deposition of ECM in damaged lungs. Therefore, suppression of abnormal ECM is another goal of silicosis therapy in addition to reducing the inflammatory response. In our study, BIC down-regulated α-SMA in epithelial cells and fibroblasts and up-regulated E-cadherin in both in vivo and in vitro silicosis models, leading to reduced synthesis of ECM components, indicating that inhibition of EMT and FMT processes by BIC could present other modes of action against silicosis, rather than the simple subsequent response of inflammation blockade.

**Fig. 10:**
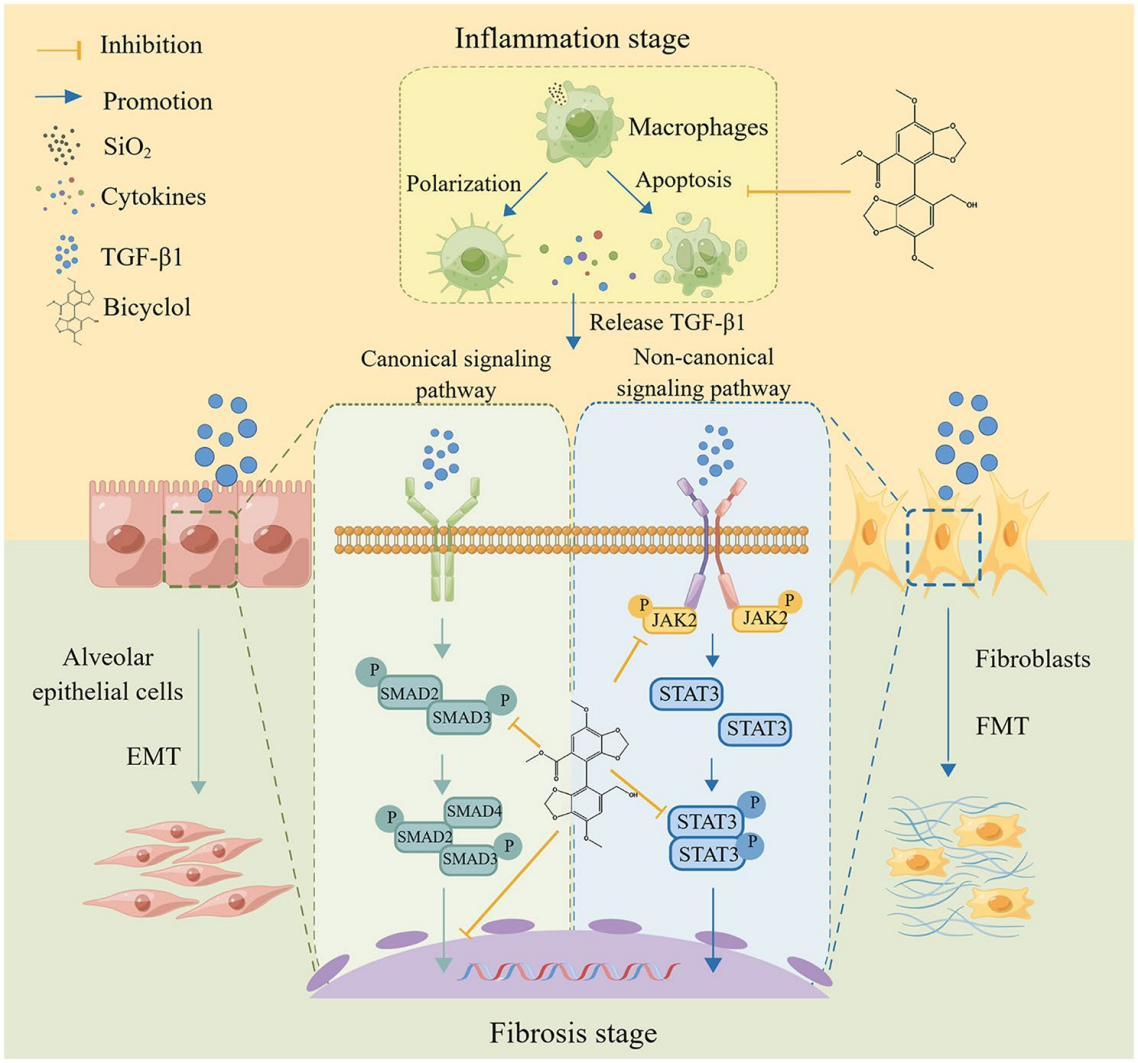
Schematic representation of the protective effects of BIC against SiO_2_-induced fibrosis. BIC blocks the polarization and apoptosis of macrophages stimulated by SiO_2_ particles and suppresses secretion of inflammatory cytokines, in particular, TGF-β1. BIC blocks the canonical TGF-β1 signaling pathway via effects on phosphorylation and nuclear translocation of both SMAD2 and SMAD3, resulting in inhibition of the EMT process in epithelial cells. On the other hand, BIC suppresses phosphorylation of JAK2 and STAT3 induced by TGF-β1 in a SMAD-independent (non-canonical signaling pathway) manner to inhibit FMT in fibroblasts, which reduces migration to damaged alveoli and synthesis of ECM components, ultimately resulting in reduced deposition of ECM and delay of fibrosis induced by SiO_2_ particles

In summary, our findings highlight the significant therapeutic effects of BIC against silicosis and provide valuable insights into the underlying molecular mechanisms. BIC attenuated SiO_2_-induced inflammation via acting on macrophages, leading to reduced secretion of inflammatory cytokines, in particular, TGF-β1. During the fibrotic stage, BIC targeted TGF-β1 to block both canonical and non-canonical signaling pathways in epithelial cells and fibroblasts and inhibited the EMT and FMT processes, followed by suppression of the synthesis and deposition of ECM components, ultimately resulting in delayed fibrosis progression (Fig. 10). Our collective findings provide an experimental basis for the clinical application of BIC in silicosis.

## Conclusion

We conclude that BIC reduces SiO_2_-induced pulmonary fibrosis by inhibiting the inflammatory fibrotic responses in silicosis via the TGF-β1/SMAD2/3 and JAK2/STAT3 signaling pathways. Silicosis has a severe adverse health, economic, and social impacts on workers on a global scale, and owing to limited therapeutic options, its management poses a considerable challenge. Numerous studies have confirmed that silicosis is significantly associated with the inflammatory response. However, anti-inflammatory therapy alone is not sufficient to delay the progression of fibrosis and may even lead to poorer clinical outcomes in patients with fibrotic lung disorders. BIC may therefore serve as a novel candidate agent with potential utility in treating silica-associated inflammation and preventing the long-term consequences of resulting fibrosis. Our collective findings provide a platform for exploring further applications of BIC in clinical therapy for silicosis and other occupational lung diseases.

### Electronic supplementary material

Below is the link to the electronic supplementary material.

Supplementary Material 1

Supplementary Material 2

## Data Availability

All datasets used and/or analyzed supporting the conclusions are available from the corresponding author upon reasonable request.

## Abbreviations

AECs: Alveolar epithelial cells
AMs: Alveolar macrophages
BALF: Bronchoalveolar lavage fluid
BIC: Bicyclol
CMC-Na: Carboxymethyl cellulose sodium
COL1A1: Collagen 1A1
COL3A1: Collagen 3A1
Crs: Compliance
CS: Crystalline Silica
Cst: Static compliance
CVF: Collagen volume fraction
ECM: Extracellular matrix
EMT: Epithelial–mesenchymal transition
Ers: Elastance
FBS: Fetal bovine serum
FEFx: Forced expiratory flow at x second
FEVx: Forced expiratory volume at x second
FMT: Fibroblast-Myofibroblast Transition
FN1: Fibronectin 1
FVC: Forced vital capacity
GAPDH: Glyceraldehyde-3-phophate dehydrogenase
HYP: Hydroxyproline
IC: Inspiratory capacity
IF: Immunofluorescence
IHC: Immunohistochemistry
ILDs: Interstitial lung diseases
IPF: Idiopathic pulmonary fibrosis
NPFE: Negative pressure forced expiration
NS: Normal saline
PFTs: Pulmonary function tests
Rn: Newtonian resistance
Rrs: Resistance
SiO_2_: Silicon dioxide
TCM: Traditional Chinese medicine
TPEF: Time to peak expiratory flow
α-SMA: Alpha-smooth muscle actin
