# Supplementary material for: Bicyclol attenuates pulmonary fibrosis with silicosis via both canonical and non-canonical TGF-β1 signaling pathways
Source: J Transl Med. 2024 Jul 26;22:682. doi: 10.1186/s12967-024-05399-x (PMC11282674; doi:10.1186/s12967-024-05399-x)
Supplement: Supplementary file 1 — Supplementary Material 1 [file 12967_2024_5399_MOESM1_ESM.docx]

***Supplementary Information***

# 1. Supplementary Materials and Methods

## 1.1 Histologic analysis

Lung tissues were fixed overnight in 4% paraformaldehyde, then embedded in paraffin. HE, Masson, TUNEL staining, immunohistochemistry (IHC), and immunofluorescence (IF) were performed by Servicebio (Wuhan Servicebio Technology Co., Ltd., Wuhan, China), according to manufacturer’s recommendations. A concentration of 1:50 of E-cadherin and α-SMA was recommended for IF. And the lung sections were evaluated for immunohistochemical localization of TGF-β1 and SOCS3. All images were visualized. Szapiel score (Table S1) [1], Ashcroft score, (Table S2) [2]and collagen volume fraction (CVF) were annotated by ImageJ software, while cell spacer thickness[3] annotated semi-quantitatively by Image-Pro Plus 5.1 software.

## 1.2 Hydroxyproline assay

The measurement of hydroxyproline (HYP) was detected with a HYP measurement kit (A030-2-1, Nanjing Jiancheng Bioengineering Institue, China) rigorously based on the manufacturer’s instructions. Approximately 30 mg (wet weight) lung tissues were taken for the detection. The absorbance at 560 nm was measured and the HYP concentration in tissues was determined against the standards.

## 1.3 ELISA

ELISA kits for the detection of IL-1β, IL-6, TNF-α, and TGF-β1 were obtained from Elabscience (Elabscience Biotechnology Co., Ltd, China). The levels of the four cytokines in lung tissues, serum, BALF and cell supernatants were detected according to the manufacturer’s instructions, respectively.

## 1.4 Protein extraction and Western blot analysis

The total proteins were extracted using RIPA lysis buffer (89900, Thermo Pierce, Rockford, USA) from lung tissues and cells, and then measured using a BCA Protein Assay Kit (23227, Thermo Pierce, Rockford, USA). Protein samples were separated on SDS-PAGE gels and transferred to PVDF membranes. After blocking with 7 % skimmed milk, the membranes were incubated with the primary antibodies overnight at 4°C. The membranes were washed in Tris-buffered saline with Tween-20 and then incubated with the secondary antibody for 2 h at room temperature. The membranes were washed again in Tris-buffered saline with Tween-20 and the bands were imaged using the Chemidoc XRS+ electrophoretic imaging system (Bio-Rad, Hercules, CA, USA), in the addition of Immobilon Western Chemiluminescent HRP Substrate (Millipore, Billerica, USA). Density scanning of each protein band was performed using Image Lab software (Bio-Rad, Hercules, CA, USA). The primary antibodies were used as Table S3.

## 1.5 RNA extraction and real-time PCR assays

Total RNA was extracted from tissues and cells with TRIzol ’reagent, and then reversely transcribed into cDNA. The conventional quantitative PCR was done using SYBR reagent (CW2601, CWBIO, China). Glyceraldehyde-3-phophate dehydrogenase (GAPDH) was used as an internal control. Data analysis was performed using the 2^-ΔΔCt^ method. Primers for qPCR are shown in Table S4.

## 1.6 Flow cytometry assay

For apoptosis detection, an apoptosis detection kit (C1062S, Beyotime, China) was used following the manufacturer’s instructions. Shortly, cells treated with SiO_2_ and BIC previously were collected and incubated with 5 μl FITC Annexin V and 10 μl PI for 20 min in dark. For cell surface marker detection, pre-treated RAW264.7 cells were collected and incubated with CD16/CD32 (0160289, BD, USA) for 10 min at room temperature, firstly. Then, the cells were washed with PBS and incubated with PE anti-mouse F4/80 (123110, Biolegend, USA), APC anti-mouse CD163 (155306, Biolegend, USA) and PE-Cy7 anti-mouse CD86 (105031, Biolegend, USA) antibodies in dark at room temperature for 30 min. The incubated cells were washed and resuspended in PBS, then the cells were analyzed by FACS (BD Bioscience, USA).

## 1.7 Cell proliferation assay

The Cell Counting Kit-8 (C0038, Beyotime, China) was used to detect cell proliferation according to the manufacturer's instructions. Briefly, 5×10^3^cells per well in triplicate were seeded into 96-well plates, and incubated at 37 °C and 5% CO_2_ overnight. Then, cells were treated with SiO_2_ and different concentrations of BIC (0, 50, 100, 150, 200 nmol/L). Assessment of cell growth was performed by the incubation of 10 μL CCK-8 at 37 °C for 2 h. Absorbance was measured at 490 nm by a 96-well multimode plate reader (BioTek, USA).

## 1.8 Wound healing assay

NIH-3T3 cells were seeded into 6-well plates and cultured with 2% FBS to diminish the influence of cell proliferation. A straight line was scratched on the monolayer cell using a sterile 20 μL pipette tip. Floating cells were then removed by washing with PBS. Wound gaps were captured by microscope.

## 1.9 Transwell migration assay

Transwell migration assay was performed using 24-well Boyden chambers (3422, Corning Incorporated, Corning, NY, USA). Briefly, 1×10^5^ NIH-3T3 cells in serum-free medium were seeded into the upper portion of a chamber. The lower chambers were filled with complete medium and either TGF-β1 (10 ng/mL) or the supernatant of SiO_2_-treated RAW264.7. All the cells cultured with different concentrations of BIC (0, 50, 100, 150, 200 nmol/L). 12 h after incubation, non-migrated cells in upper chambers were removed by cotton swabs. The migrated cells were fixed, stained with 0.5% crystal violet (C0121, Beyotime, China), and then photographed.

# 2. Supplementary Tables and Figures

## Supplementary Tables

**Table S1. Szapiel scoring rules for alveolar inflammation.**

| Lever | Morphological analysis under the microscope | Scored |
| --- | --- | --- |
| 1 | No alveolar inflammation | 0 |
| 2 | Mild alveolitis, with an area less than 20% of the lung | 1 |
| 3 | Moderate alveolar inflammation, involving 20–50% of the lung | 2 |
| 4 | Severe alveolar inflammation, involving more than 50% of the lung | 3 |

**Table S2. Ashcroft scoring rules for fibrosis.**

| Lever | Morphological analysis under the microscope | Scored |
| --- | --- | --- |
| 0 | Normal | 0 |
| 1 | Partially enlarged alveoli, alveolar septum thickened slightly | 1 |
| 2 | Alveolar septum thickened moderately, without damage to the lung architecture | 2 |
| 3 | Alveolar septum thickened moderately, and increased fibrotic tissue increased | 3 |
| 4 | The area of fibrous tissue mass is less than 10% of the lung, with mild lung structural damage | 4 |
| 5 | The area of fibrous tissue mass is 10–50% of the lung, and with pulmonary structural damage | 5 |
| 6 | The area of fibrous tissue mass is more than 50% of the lung, and with obvious pulmonary structural damage | 6 |
| 7 | Severe lung damage, large areas of fibrosis, honeycomb lung | 7 |
| 8 | Full field fibrous tissue | 8 |

**Table S3. The information of antibody.**

| Antibodies | Source | Identifier |
| --- | --- | --- |
| STAT3 | Cell Signaling Technology | 9139 |
| P-STAT3 | Cell Signaling Technology | 9145 |
| JAK2 | Cell Signaling Technology | 3230 |
| P-JAK2 | Cell Signaling Technology | 3776 |
| SOCS3 | Abcam | ab280884 |
| SMAD2 | Cell Signaling Technology | 5339 |
| P-SMAD2 | Cell Signaling Technology | 18338 |
| SMAD3 | Cell Signaling Technology | 9523 |
| P-SMAD3 | Cell Signaling Technology | 9520 |
| TGF-β1 | Abcam | Ab215715 |
| E-cadherin | Cell Signaling Technology | 14472 |
| α-SMA | Affinity | AF1032 |
| COL1A1 | Affinity | AF7001 |
| COL3A1 | Affinity | AF0136 |
| FN1 | Cell Signaling Technology | 63379 |
| iNOS | Abcam | Ab15323 |
| Arg1 | Abcam | Ab233548 |
| Pro-Caspase-3 | Affinity | AF6311 |
| Cleaved-Caspase-3 | Affinity | AF7022 |
| GAPDH | Abcam | ab263962 |
| Lamin B1 | Cell Signaling Technology | 12586 |

**Table S4. The forward and reverse primers for real-time PCR.**

| Gene | Forward primer | Reverse primer |
| --- | --- | --- |
| Mouse-IL-1β | AGCCAGAGTCCTTCAGAGAGAT | GAGAGCATTGGAAATTGGGGT |
| Mouse-IL-6 | CAGCTGGAGAGTGTGGATCC | TGCTTGTGAGGTGCTGATGT |
| Mouse-TGF-β1 | ATTGGCCAGCATCCATCTCTTG | GTGCAGGATGCATTGCTGAC |
| Mouse- TNF-α | CACAGAAAGCATGATCCGCG | ACTGATGAGAGGGAGGCCAT |
| Mouse-COL1A1 | TTCTCCTGGCAAAGACGGAC | CGGCCACCATCTTGAGACTT |
| Mouse-COL3A1 | ACGTAAGCACTGGTGGACAG | CAGGAGGGCCATAGCTGAAC |
| Mouse-FN1 | ATGAGAAGCCTGGATCCCCT | GGAAGGGTAACCAGTTGGGG |
| Mouse-GAPDH | CATCAAGAAGGTGGTG | CCTGTTGCTGTAGCC |
| Rat-IL-1β | GTGCTGTCTGACCCATGTGA | GATTCTTCCCCTTGAGGCCC |
| Rat-IL-6 | TCTGGGAAATCGTGGAAATGAGA | TCTCTCTGAAGGACTCTGGCT |
| Rat-TGF-β1 | AGCTCCACAGAGAAGAACTGC | TCATGTTGGACAACTGCTCC |
| Rat-TNF-α | GCCTCCTCTCTGCCATCAAG | CTCCAAAGTAGACCTGCCCG |
| Rat-COL1A1 | GGAGAGAGCATGACCGATGG | GGGACTTCTTGAGGTTGCCA |
| Rat-COL3A1 | CTTCTCACCCTGCTTCACCC | GGGCAGTCTAGTGGCTCATC |
| Rat-FN1 | ATGAGAAGCCTGGATCCCCT | CAGTTGGGGAAGCTCATCTGT |
| Rat-GAPDH | GGCTGCCTTCTCTTGTGAC | TCCCGTTGATGACCAGCTTC |

## Supplementary figures


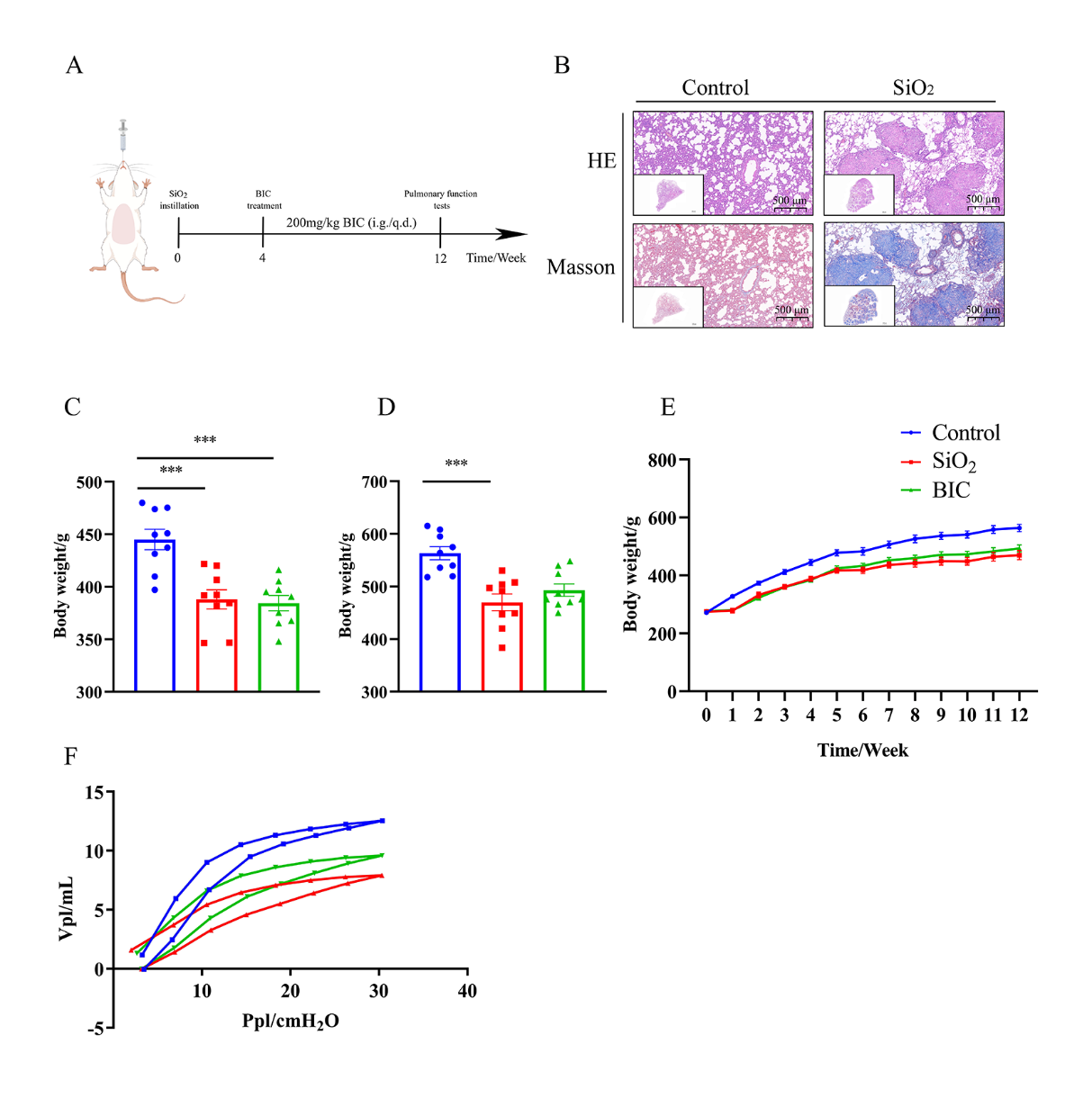


**Figure S1. BIC therapeutic treatment ameliorates impaired lung function in silicosis rats. A**. Schematic of BIC administration in a therapeutic silicosis rat model. There are three groups (control, SiO_2_, BIC) in the following experiments. Control group treated with NS and vehicle; SiO_2_ group installed with SiO_2_ and vehicle by gavage; BIC group installed with SiO_2_ and BIC by gavage. **B.** Representative HE (above) or Masson (below) photographs of lung sections from the silicotic rats and control rats at 4 weeks after SiO_2_ or NS instillation. Up: 1🞨, down: 10🞨. **C-D**. Body weight of rats at 4 weeks (**C**), 12 weeks (**D**) after SiO_2_ or NS instillation. **E.** Weight growth of each group of rats during the whole experiment. **F**. Mean PV loops of each group. All data are represented as mean ± SEM. Graphs in **C-F** share the same symbols (blue for Control, red for SiO_2_ and green for BIC). **p* < 0.05, ***p*<0.01, and ****p* < 0.001 compared to the SiO_2_ group. n =9 per group.


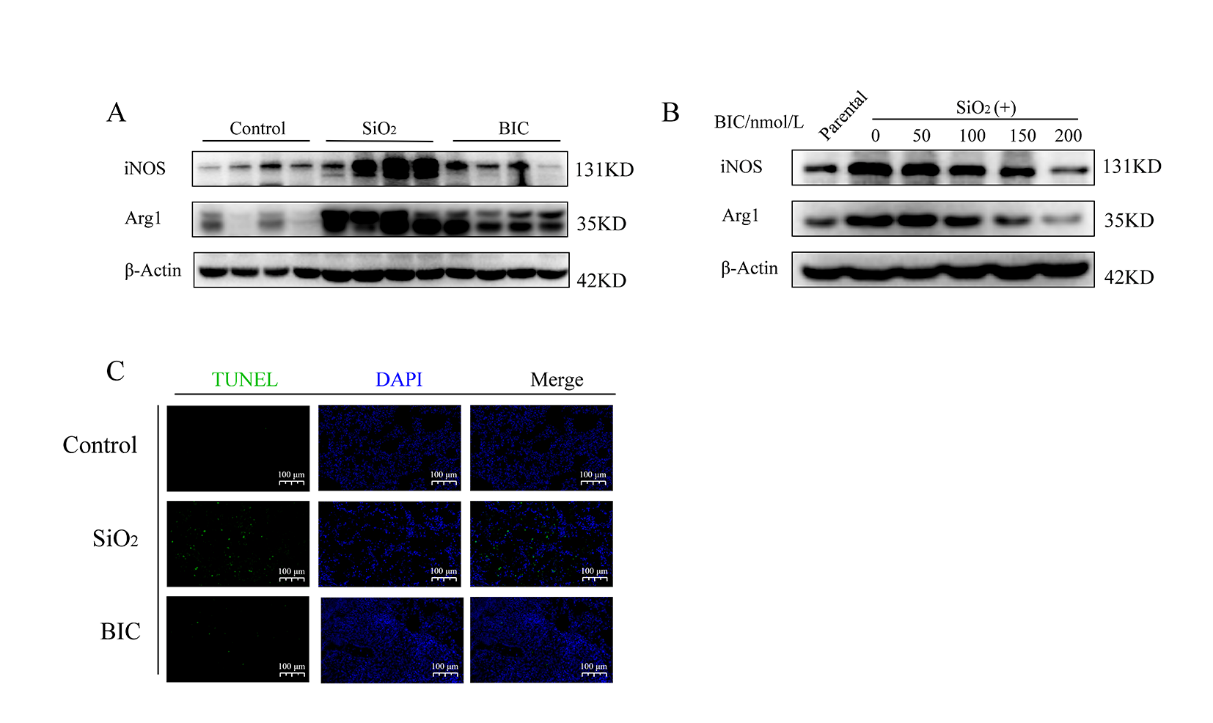


**Figure S2. BIC regulates the polarization and apoptosis of macrophages. A-B.** Western blot analysis of the expression of iNOS and Arg1 in both lung tissues (**A**) and RAW264.7 cells (**B**). **C.** The induction of apoptosis *in vivo* was evaluated by TUNEL staining (50🞨) using lung tissues extracted from silicotic rats.


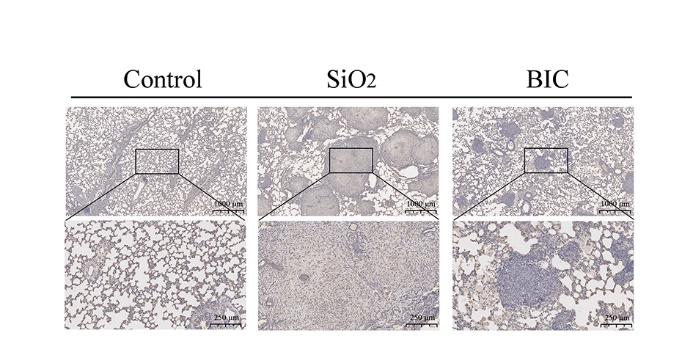


**Figure S3. BIC reduces the expression of SOCS3.** The expression of SOCS3 in lung sections by IHC staining, up: 5🞨, down: 20🞨.


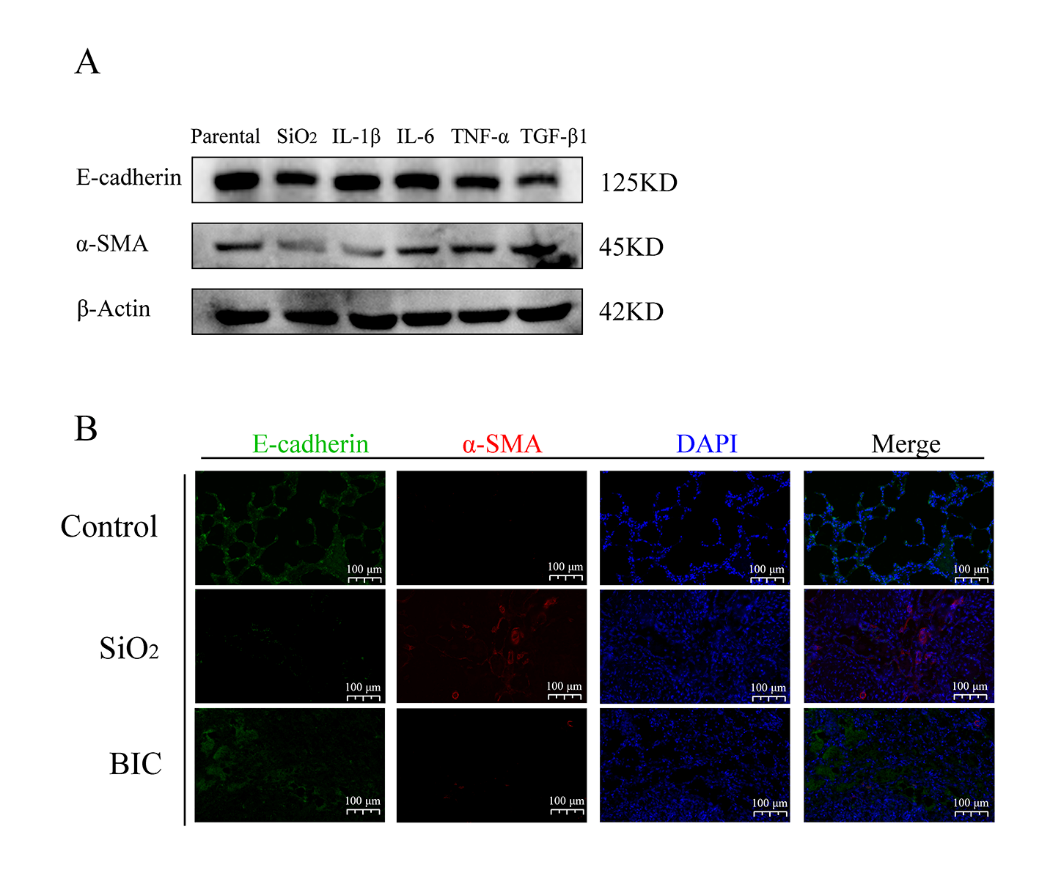


**Figure S4. BIC suppresses the progress of EMT by TGF-β1/SMAD2/3. A.** TC-1 cells were treated with SiO_2_ (100 μg/ml), IL-1β (100 ng/mL), IL-6 (100 ng/mL), TNF-α (100 ng/mL), and TGF-β1 (10 ng/mL) for 48 h, respectively. The expression of EMT markers (E-cadherin and α-SMA) were detected using western blot assay. **B**. Representative photos of E-cadherin (green) and α-SMA (red) in silicotic lung tissues by IF staining (50🞨).


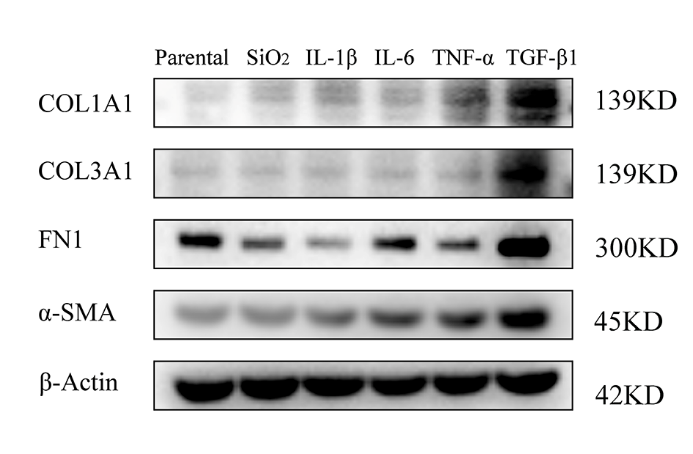


**Figure S5. TGF-β1 secreted by macrophages plays a major role in driving FMT.** NIH-3T3 cells were treated with SiO_2_ (100 μg/ml), IL-1β (100 ng/mL), IL-6 (100 ng/mL), TNF-α (100 ng/mL), and TGF-β1 (10 ng/mL) for 12 h. The FMT marker COL1A1, COL3A1, FN1 and α-SMA were analyzed in these cells.

# Supplementary References

[1] S.V. Szapiel, N.A. Elson, J.D. Fulmer, G.W. Hunninghake, R.G. Crystal, Bleomycin-induced interstitial pulmonary disease in the nude, athymic mouse, Am Rev Respir Dis 120(4) (1979) 893-9.

[2] T. Ashcroft, J.M. Simpson, V. Timbrell, Simple method of estimating severity of pulmonary fibrosis on a numerical scale, J Clin Pathol 41(4) (1988) 467-70.

[3] T.C. Beller, D.S. Friend, A. Maekawa, B.K. Lam, K.F. Austen, Y. Kanaoka, Cysteinyl leukotriene 1 receptor controls the severity of chronic pulmonary inflammation and fibrosis, Proc Natl Acad Sci U S A 101(9) (2004) 3047-52.
