# Supplementary material for: Bicyclol attenuates pulmonary fibrosis with silicosis via both canonical and non-canonical TGF-β1 signaling pathways
Source: J Transl Med. 2024 Jul 26;22:682. doi: 10.1186/s12967-024-05399-x (PMC11282674; doi:10.1186/s12967-024-05399-x)
Supplement: Supplementary file 2 — Supplementary Material 2 [file 12967_2024_5399_MOESM2_ESM.docx]

RE：Manuscript: JTRM-D-24-03578

Dear editor,

Thank you so much for reviewing our manuscript entitled “Bicyclol Attenuates Pulmonary Fibrosis with Silicosis via both Canonical and Non-canonical TGF-β1 signaling pathways”. The comments from the reviewers are all valuable and very helpful for improving the manuscript. We have revised the manuscript and figures carefully according to the reviewers’ comments. Revised portions are marked in red in the re-submitted manuscript. Listed below are our responses to the reviewers’ comments and the changes we have made. If there are any incorrect answers or questions in the manuscript, please do not hesitate to let us know. We hope this revised manuscript is now acceptable for publication on *Journal of Translational Medicine*.

Sincerely yours,

Tong-Tong Liu; Yun Zhan, Ph D

Professor of Pharmacology

State Key Laboratory of Bioactive Substances and Function of Natural Medicine

Institute of Materia Medica

Chinese Academy of Medical Sciences & Peking Union Medical College

Beijing 100050, P.R.CHINA

Tel & Fax: +86-10-63017906, E-mail: zhanyun@imm.ac.cn

**Point-by-point response to reviewers**

**Reviewer 1:**

**Major comments:**

**In this paper, the authors established a silicotic rat model and three cell models to evaluate the potential therapeutic effects and explore the molecular mechanisms of BIC. The results demonstrated that BIC ameliorated inflammatory cytokine secretion and consequently inhibited FMT and EMT via TGF-β1 canonical and non-canonical pathways, ultimately resulting in reduction of aberrant collagen deposition and slower progression of silicosis, supporting its potential as a novel therapeutic agent. BIC significantly improved static compliance of lung and expiratory and inspiratory capacity of silica-induced rats. This article provides new ideas for the mechanism study and treatment of silicosis fibrosis. However, there are still some problems that need to be improved.**

**1. In the Method section, how is the concentration of silica determined, and is there any literature to support it?**

**The author’s response:**

In our research, rats were intratracheally instilled with 1 mL sterile SiO_2_ suspension (100 mg/mL). There are many literatures support the method like Jiao et.al [1] and Li et. al [2]. And RAW264.7 cells were stimulated with SiO_2_ suspension (100 μg/mL), which was also supported by some researches [3].

[1] Jiao J, Li L, Yao W, et al. Influence of Silica Exposure for Lung Silicosis Rat [J]. Dis Markers, 2021, 2021: 6268091.

[2] Li J, Yao W, Hou JY, et al. The Role of Fibrocyte in the Pathogenesis of Silicosis [J]. Biomed Environ Sci, 2018, 31: 311-316.

[3] Li J, Yao W, Hou JY, et al. Crystalline Silica Promotes Rat Fibrocyte Differentiation in Vitro, and Fibrocytes Participate in Silicosis in Vivo [J]. Biomed Environ Sci, 2017, 30: 649-660.

**2. M1 macrophages are thought to have pro-inflammatory effects, while M2 macrophages mainly play a role in inhibiting inflammation and promoting tissue repair. However, in the Results section, you mentioned: "BIC treatment led to a dose-dependent decrease in the ratios of CD86+and CD163+cells in RAW264.7 macrophages activated with SiO_2_, indicating inhibitory effects on macrophage polarization to both M1 and M2 types." Is BIC an anti-inflammatory or pro-inflammatory effect in this model?**

**The author’s response:**

In the early stage of silicosis, M1 macrophages are stimulated, which promotes inflammation primarily through the production of the pro-inflammatory cytokines IL-1β, TNF-α, and IL-6. Later, M2 macrophages are induced to secrete the pro-fibrotic cytokines IL-10 and TGF-β1, which promotes tissue repair and fibrosis in the late stage of silicosis [1]. Therefore, inhibiting macrophage polarization can reduce the symptoms of silicosis. RAW264.7 cells were stimulated with SiO_2_ for 8 h and the ratio of M1/M2 polarization was detected via flow cytometry. BIC treatment led to a dose-dependent decrease in the ratio of CD86+ especially (from 14.7% to 5.26%), indicating the anti-inflammatory effect of BIC. And BIC also downregulated the ratio of M2 (from 3.61% to 1.82%) and decreased the concentration of TGF-β1, which indicated the anti-fibrosis effect of BIC. However, it is difficult to distinguish the stages of inflammation and fibrosis strictly, the inhibition of both the M1 and M2 polarization by BIC could be observed, as well as the of decrease of the secretion of IL-1β, TNF-α, IL-6, and TGF-β1, suggesting both the anti-inflammatory and anti-fibrosis effects of BIC.

[1] Carneiro PJ, Clevelario AL, Padilha GA, Silva JD, Kitoko JZ, Olsen PC, Capelozzi VL, Rocco PR, Cruz FF. Bosutinib Therapy Ameliorates Lung Inflammation and Fibrosis in Experimental Silicosis. Front Physiol. 2017 Mar 15;8:159.

Figure: the ratios of macrophages polarization of parental group, 0 nmol/ml BIC group and 200 nmol/ml BIC group.

**3. In Result 3.5, "Earlier reports have demonstrated that TGF-β1 activates SMAD2/3 for binding to promoters of fibrosis genes and modulates progression of EMT." Relevant literature should be supplemented.**

**The author’s response:**

Thank you for your feedback. We have added the reference in manuscript.

Page11, line2-4:

Earlier reports have demonstrated that TGF-β1 activates SMAD2/3 for binding to promoters of fibrosis genes and modulates progression of EMT [24, 25].

[24] J.H. Lee, J. Massagué, TGF-β in developmental and fibrogenic EMTs, Semin Cancer Biol 86(Pt 2) (2022) 136-145.

[25] H.H. Hu, D.Q. Chen, Y.N. Wang, Y.L. Feng, G. Cao, N.D. Vaziri, Y.Y. Zhao, New insights into TGF-β/Smad signaling in tissue fibrosis, Chem Biol Interact 292 (2018) 76-83.

**4. The explanatory content in the Results section might be better moved to the Discussion section.**

**The author’s response:**

Thank you for your advice. We have carefully reviewed the original manuscript and made some necessary revisions to improve its coherence. All the revisions were marked red. Specifically, in Result 3.2, we moved the introduction of BIC to Discussion section (page14, line10) and simplified the description. In Result 3.3, we shifted the sentences of M1/M2 polarization and aberrant apoptosis induced by SiO_2_ to Discussion section (page14, line19). In Result 3.4, we deleted the description of cytokines secreted by M1/M2 macrophage and moved it to Discussion section (page14, line21), which made our article more clarity. In Result 3.5 and 3.7, we deleted a few sentences of FMT and EMT, which were repetitive and have been discussed in our manuscript (page17 line3-10). All changes had been marked in red in re-submitted manuscript.

**5. In Figure 2H, the expression of COL1A1 in the fourth well of the control group was higher than that of the other wells, but the error bars in the quantification plot were not high? In addition, the expression of COL1A1 in the control group was generally higher than that in the BIC group, so why its expression lower in the quantification figure than in the BIC group?**

**The author’s response:**

All of the lung tissues (n=9) were tested by Western Blot and quantitative analysis. And the pictures showing in our manuscript is the representative image of 4 animals.

Figure 2H: Representative images of Western blots (left) of COL1A1, COL3A1, and FN1 in lung tissue. The picture we were showing in our manuscript were the representative image of 4 animals.

And the picture below were their original western blots.

The pictures of quantitative protein expression in our manuscript were all of the animal (n=9). We tested the protein level of control group, SiO_2_ group and BIC group by Western blot and analyzed the density scanning by Image J software.

And the pictures below were original western blots.

**6. There are some minor issues here:**

**1) Page 5 Line 22-26: References should be inserted here.**

**2) There is no scale bar in the diagram of pathological morphological changes, and it is recommended to add it.**

**3) The WB plot suggests adding the molecular weight of the corresponding protein.**

**4) In page 15, line 25, PFTs' relevant literature should be supplemented.**

**The author’s response:**

1) As suggested by the reviewer, we have added more references to support this idea and marked this sentence in red.

In manuscript:

The formation and development of pulmonary fibrosis is a profoundly complex biological process. Inflammation is one of the most common causes of silicosis in which macrophages play an important role [4]. Alveolar macrophages (AMs) that recognize and engulf silicon dust are transported to the lung interstitium and differentiate into activated interstitial macrophages [5]. Subsequently, these macrophages undergo dysregulated polarization, with secretion of abundant cytokines such as IL-1β, IL-6, TNF-α and TGF-β1, further promoting inflammation and progression of silicosis [6].

[4] Du S, Li C, Lu Y, Lei X, Zhang Y, Li S, Liu F, Chen Y, Weng D, Chen J, Dioscin Alleviates Crystalline Silica-Induced Pulmonary Inflammation and Fibrosis through Promoting Alveolar Macrophage Autophagy. Theranostics 2019;9:1878-1892.

[5] Liu TT, Sun HF, Han YX, Zhan Y, Jiang JD, The role of inflammation in silicosis. Front Pharmacol 2024;15:1362509.

[6] Adamcakova J, Mokra D, New Insights into Pathomechanisms and Treatment Possibilities for Lung Silicosis. Int J Mol Sci 2021;22:

2) Thank you for your reminder, we have added the scale bar in the diagram of pathological morphological changes in figure 2 and supplementary figure S1-S4.

Figure 2A: Representative photographs of lung sections from the three treatment groups of the silicosis rat model stained with either HE (above) or Masson (below) at 1🞨 and 10🞨 magnification.

Figure S1B: Representative HE (above) or Masson (below) photographs of lung sections from the silicotic rats and control rats at 4 weeks after SiO_2_ or NS instillation. Up: 1🞨, down: 10🞨.

Figure S2C: The induction of apoptosis *in vivo* was evaluated by TUNEL staining (50🞨) using lung tissues extracted from silicotic rats.

Figure S3: The expression of SOCS3 in lung sections by IHC staining, up: 5🞨, down: 20🞨.

Figure S4B: Representative photos of E-cadherin (green) and α-SMA (red) in silicotic lung tissues by IF staining (50🞨).

3) We have added the molecular weight of the corresponding protein in figure 2-8 and figure S2-5.

Figure 2H:

Figure 3C:

Figure 4C-D:

Figure 5:

Figure 6B-C:

Figure 8B-C:

Figure S2A-B:

Figure S4A:

Figure S5:

4) We have supplemented related literatures about PFTs in our manuscript (page 8 and 13, reference 19). As demonstrated in an excellent research by Devos et al [1], pulmonary function tests (PFTs) are important when studying respiratory disease models. In their article, bleomycin was used to induce mice fibrosis. And we can see a characteristic downward shift of PV loop of mice with fibrosis compared to naive control mice, which implies an increased stiffness of the lungs. And mice with fibrosis showed a significant decrease in IC and Cst compared to naive controls. All of the changes consistent with our silicotic rats. And they also showed that mice with fibrosis showed a significantly reduced FEV0.1 and FVC, as well as a normal FEV0.1/FVC ratio. In our silicotic model, SiO_2_ induced a significant decrease in the FEV0.2/FVC ratio and FEF0.1 and increase in TPEF, which were not same as mice. The different pressures of instrument used in rats and mice may account for some of the differences observed.

[1] Devos FC, Maaske A, Robichaud A, et al. Forced expiration measurements in mouse models of obstructive and restrictive lung diseases. Respir Res. 2017;18(1):123. Published 2017 Jun 19. doi:10.1186/s12931-017-0610-1

**Reviewer #2:**

**Major comments：**

**The research idea is clear and the overall mechanism is relatively perfect.**

**The author’s response:**

We sincerely thank the reviewer for the valuable and positive feedback of our manuscript.
